# ﻿A case study on *Anemone hortensis* L. (Ranunculaceae): the misapplication of *A. pavonina* Lam. and *A. fulgens* (DC) Rchb. has led to taxonomic and nomenclatural confusion

**DOI:** 10.3897/phytokeys.263.158703

**Published:** 2025-09-24

**Authors:** James A. Compton

**Affiliations:** 1 Spilsbury Farm, Salisbury, SP36RU, UK Spilsbury Farm Salisbury United Kingdom

**Keywords:** *
Anemone
*, misapplication, nomenclature, typification

## Abstract

*Anemone hortensis* L. was typified on a specimen with red flowers and obovate tepals cultivated in the Netherlands, originally introduced from the eastern Mediterranean. This red-flowered variant of the species has been in cultivation in northern Europe since the 16^th^ century and is now widely naturalised in northwest Italy and southeast and southwest France. *Anemone fulgens* (DC.) Rchb., also with red flowers, is shown to be a synonym of *A. hortensis*. *Anemone pavonina* Lam. is revealed to have been based on a teratological red-flowered specimen of *A. hortensis* with multiple tepals and is recognised here as A. hortensis var. pavonina (Lam.) Gren. and Godr. Plants across the range of the species with lanceolate, frequently lilac or purple tepals, which are often incorrectly attributed to *A. pavonina*, are shown to be A. hortensis var. stellata Gren. and Godr. Plants endemic to Crete and its adjacent islands, with elliptic white or pale pinkish flowers, are recognised as A. hortensis var. heldreichii (Boiss.) Halácsy. A total of 16 lectotypes and one neotype are designated, and a key to the varieties within *A. hortensis* is provided along with synonymic conspectuses. The new combination A. hortensis f. regina (Risso) J. Compton is made to identify plants with red or purple flowers having a zone of yellow or white at the base of each tepal, collectively forming a pale central circle within the flower.

## ﻿Introduction

The temperate to subtropical genus *Anemone* L. (Ranunculaceae), with c. 109 species worldwide, includes the well-known red-flowering Mediterranean anemones. These belong to either one of two species: the poppy anemone, *Anemone coronaria* L., or the garden anemone, *A. hortensis* L., as both species produce a range of flower colours that include a vibrant red. It is unclear whether Publius Ovidius Naso, better known as Ovid, was referring to *A. coronaria* or *A. hortensis* in the legend from the poem *Metamorphoses* penned c. AD 8. In the poem the mortal Adonis, who had been gored by a boar, was having nectar poured from an ewer onto him by the mourning Greek/Cypriot goddess Aphrodite [Roman Venus], whose tears mixed with Adonis’s blood as he lay dying. From where the tears fell onto the barren ground, red-flowered anemones emerged. The anemone in the myth of Aphrodite and Adonis could refer to either species, native across much of the eastern Mediterranean. Although only one of several Renaissance paintings depicting this tragedy, Nicolas Poussin’s painting “Venus Lamenting Over Adonis” c. 1630, now in the Musée des Beaux-Arts de Caen in France, recalls the scene of Adonis’s death. Poussin’s painting was possibly based on the engraving by Pieter van der Borcht (c. 1530–1608), entitled “Adonis in Anemonem Florem”, in the first illustrated edition of Ovid’s work “Argumentis brevioribus ex Luctatio Grammatico collectis expositae” (Anonymous 1591: 267). The botanist Carolus Clusius (1526–1609) believed it was *Anemone coronaria*, which, as his second sort, he called “Anemone tenuifolia flore polyphyllo” (Clusius 1583: 391), describing it (translated from Latin): “As for some kinds of Anemone, it is said to refer to the flower of Adonis as described by Ovid in Book 10 of *Metamorphoses*, I do not remember seeing any so far that comes closer to its description than the one we present here in the second place, for its flower has a colour similar to blood, or to the juice of an unripe pomegranate, especially when it is sweet or vinous” (Clusius 1583). However, as we shall see, it could also have been *Anemone hortensis*.

*Anemone hortensis* has been known in cultivation for hundreds of years (e.g. Dodoens 1563, 1583; Clusius 1601; C. Bauhin 1623; J. Bauhin et al. 1651). It is likely that Luigi Squalermo, better known as Anguillara (c. 1512–1570), was referring to the wild, knotty-rooted and purple-flowered *Anemone hortensis* growing throughout Dalmatia and around Bologna when he wrote about *Anemone* in his book *Semplici* (Anguillara 1561). Rembert Dodoens (1516/1517–1585) tells of its cultivation in Germany near the river Rhine in the mid-16^th^ century in the second edition of his plant book, or *Cruyde boeck*, with an illustration of the plant on the previous page (Dodoens 1563). The natural range of this species extends from southern France to southwest Turkey. The leaves of this species are trilobed, the lobes sometimes further divided, and it exhibits an involucre or collarette comprising a trio of sessile, lanceolate leaflets on the flower stem some distance below the flower and well above the basal leaves. The flowers exhibit a wide panoply of colours across the range of the species, including lilac, violet, pink, white and red; the tepals vary in number from eight to 18. One such early example of a herbarium specimen with 18 tepals is in the Herbarium of William Sherard (1659–1728) in OXF [sher-2835-b]. Sherard visited Italy as tutor to the Marquess of Tavistock in 1697 before being appointed consul for the Turkey Company in Smyrna [Izmir, Turkey] from 1703 to 1716 (Allen 2013).

**Figure 1.**
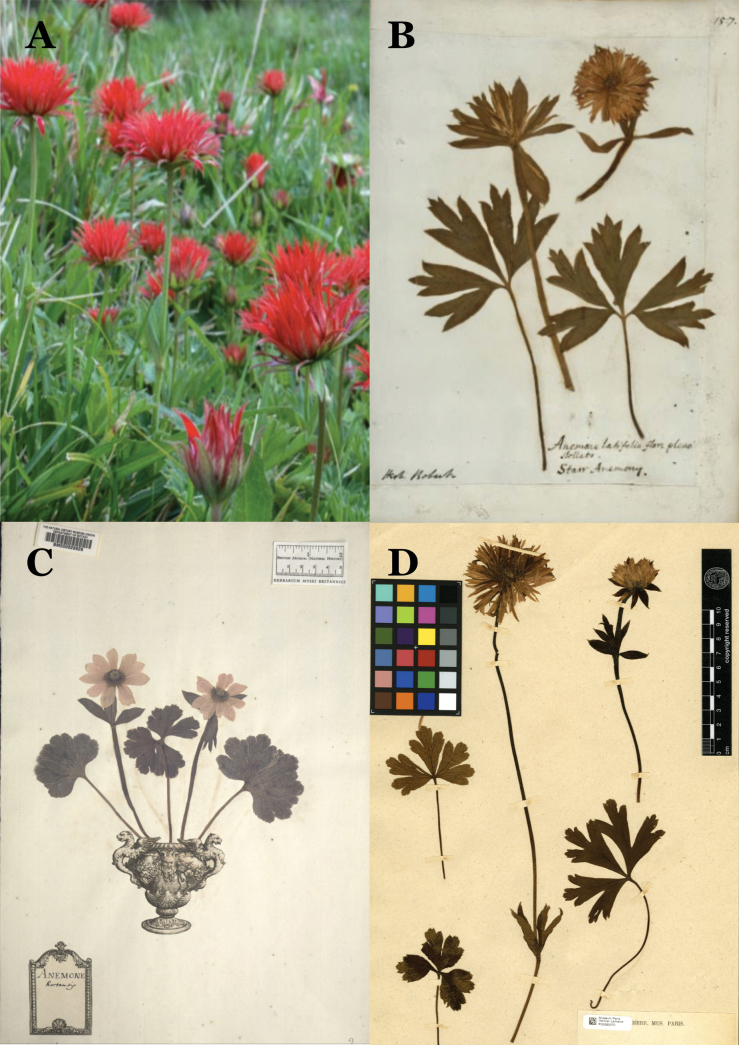
A. Anemone hortensis var. pavonina naturalised in southwest France: photo credit Pépinières du lac des Joncs, Dordogne, France; B. Jacob Bobart’s Anemone hortensis var. pavonina cultivated in the University of Oxford botanic garden c. 1680 (OXF Bjr-03-157); C. Lectotype of *Anemone hortensis* from the Clifford Herbarium (BM000628826) showing obovate tepals and remnants of reddish colouring; D. Lectotype specimen of *Anemone pavonina* (P00282070).

As with the related species *A. coronaria*, the flowers of *A. hortensis* frequently exhibit the horticulturally desirable homeotic mutation known as doubling. This is a genetic condition whereby the stamens become petaloid, forming a radiating mass of narrow tepals numbering up to 50, described in this paper as having multiple tepals or double flowers (Fig. 1A). In rare cases the outer ring of tepals can even develop leaf-like photosynthetic bracts while the central mass of tepals remains petaloid, such as in the example cultivated in the 17^th^ century in the University of Oxford Physic Garden by Jacob Bobart the younger (1641–1719) in OXF [Bjr-03-157] (Fig. 1B). The doubling of the floral parts in *Anemone* has enhanced the plant’s aesthetic appeal to horticulture in the same way that such doubling has inspired interest in camellias, carnations, daffodils, peonies, the turban buttercup (*Ranunculus asiaticus* L.) and many roses. Their appeal is exemplified by the herbalist and apothecary John Parkinson, who in the 17^th^ century figured seven different single-flowered forms of *A. hortensis* and five different double-flowered forms, all presented on a single plate (Parkinson 1629).

*Anemone coronaria* and *A. hortensis* have been shown to belong with a third species, *A. palmata* L., in Anemone sect. Anemone subsect. Anemone (Ehrendorfer et al. 2009). The three species in the subsection share several apomorphic features, notably the presence at the base of the leaf petioles of stipule-like lobes or broadened leaf-bases, an involucre of more or less connate, sessile stem-leaves, and tepals each with three to nine basal veins (Ehrendorfer et al. 2009: 345). *Anemone palmata* has pale to deep yellow flowers, frequently exhibiting a bronze colouration on the reverse of the tepals, and palmate leaves with three to seven lobes. It occurs only in the southwest Mediterranean region in Algeria, France, Morocco, Portugal, Sardinia, Sicily, Spain and Tunisia (Ehrendorfer et al. 2009). Neither *A. palmata* nor *A. coronaria* is the focus of this paper, which concerns the identity, variation and nomenclature of the extremely morphologically variable species *A. hortensis* and the assessment of plants described variously as *A. pavonina* Lam., *A. stellata* Lam. and *A. fulgens* (DC.) Rchb.

*Anemone pavonina* Lam. was described by Lamarck (Lamarck 1783; see below). It has never been typified and, as a result, has been the source of almost continual misidentification, misrepresentation and confusion, being erroneously identified in morphological and floristic works (e.g. Ulbrich 1905; Bowles 1948; Raab-Straube et al. 2014; Thorogood 2019, 2021). As a result, material under that name has frequently been used for sampling in molecular phylogenetic analyses (e.g. Hoot et al. 1994; Hoot 1995; Hoot et al. 2012; Mlinarec et al. 2016; Xiang et al. 2024). Similarly misnamed samples were used in a cytotaxonomic study of Mediterranean species of *Anemone* (Maïa and Venard 1976) and in a cytogenetic study based on chromosome morphology and heterochromatin distribution (Mlinarec et al. 2006).

The identity and wild distribution of plants named *A. fulgens*, whether as a species or in some cases as a hybrid, have similarly been the subject of long-term debate (e.g. Pritzel 1841; Bowles 1948; Maïa and Venard 1976). This name has also not been typified, and, as a result, the taxonomic status of plants assigned under that name has never been correctly ascertained.

Hitherto, the treatments of these taxa in revisions, floras and field guides have not delved comprehensively into the history and origins of either *A. hortensis*, *A. pavonina* or *A. fulgens*. Several attempts have been made to assess the differences between these taxa, but these appear to have relied solely upon previous floristic and taxonomic treatments without reference to type material (e.g. Grateloup 1820; Loret 1859; Moggridge 1864, 1874; Pons 1883; Luzzatto 1932a, b, 1933, 1934a, b; Bowles 1948; Tutin 1964; Polunin and Smythies 1973; Davis et al. 1984; Polunin 1988; Davies and Gibbons 1993; Ziman et al. 2011; Brickell 2011; Thorogood 2019, 2021).

The geographical segregation of A. hortensis into subsp. hortensis from Albania westwards to France and subsp. heldreichii (Boiss.) Rech.f., endemic to the islands of Crete and Karpathos, and the acceptance of *A. pavonina* Lam., restricted to the Aegean region including western Turkey, is followed by authoritative checklists such as Euro+Med (Raab-Straube et al. 2014). The basis for this geographic distribution originated from Ulbrich’s statement, which, however, lacked any supporting evidence (Ulbrich 1905). An alternative treatment of what has been assumed to be *A. pavonina* is at the rank of subspecies within *A. hortensis*, once again with the same purported eastern Mediterranean distribution, as recognised in, for example, “Plants of the World Online” (POWO) and the International Plant Names Index (IPNI) (https://www.ipni.org; https://powo.science.kew.org/taxon/urn:lsid:ipni.org:names:77253162-1, *Anemone fulgens*, meanwhile, is generally accepted to belong as a synonym of *A. hortensis* (e.g. IPNI (2025) and POWO 2025) or to be of hybrid origin between *A. coronaria* and *A. hortensis* (e.g. Mabberley 2017).

This paper aims to determine the precise taxonomic identity and nomenclature of plants that have been classified as *A. fulgens*, *A. hortensis*, *A. pavonina* and *A. stellata*, and to establish the infraspecific status of variation within *A. hortensis*.

## ﻿Materials and methods

These materials and methods correspond to a synthesis of the relevant collective information and data associated with the taxa which are the subject of this paper. Close examination of specimens was undertaken on material from the following herbaria: AV, B, BM, BR, E, G, K, LINN, LY, MPU, NCY, NICE, S, TLON, TO, UPS, WAG, WU. The methods employed here are undertaken under the following headings:

1. A review of historically relevant morphological treatments of the Mediterranean species of *Anemone*. The previous revisions and treatments of the studied taxa included those of Candolle (1818, 1824), Sprengel (1825), Pritzel (1841), Ulbrich (1905), Luzzatto (1932a, b, 1933, 1934a, b), Bowles (1948), Ziman et al. (2008), and Ehrendorfer et al. (2009).
2. A review of the relevant molecular and cytological research that has been undertaken on plants that have been classified with the names *A. fulgens*, *A. hortensis*, *A. pavonina* and *A. stellata*. These included detailed analyses of these taxa in the treatments of Maïa and Venard (1976), Hoot et al. (1994, 2012), Ehrendorfer and Samuel (2001), Mlinarec et al. (2006, 2016), Ehrendorfer et al. (2009), Besendorfer and Mlinarec (2013), and Xiang et al. (2024).
3. *Anemone hortensis* – an evaluation of Linnaeus’s species concept (Linnaeus 1738, 1753, 1759, 1762, 1767, 1770, 1771, 1774).
4. *Anemone pavonina* – an evaluation of Lamarck’s species concept (Lamarck 1779, 1783, 1794).
5. *Anemone stellata* – an evaluation of Lamarck’s species concept (Lamarck 1779, 1783).
6. *Anemone pavonina* var. *fulgens* – an evaluation of Candolle’s varietal concept (Candolle 1805, 1815, 1818, 1824).
7. *Anemone hortensis* var. *stellata* – an evaluation of Grenier and Godron’s varietal concept (Grenier and Godron 1847).
8. *Anemone stellata* var. *heldreichii* – an evaluation of Boissier’s varietal concept (Boissier 1849).

## ﻿Results

### ﻿Morphological review

The great Swiss botanist Augustin Pyramus de Candolle (1778–1841) described several species of *Anemone* with ovoid hairy carpels, having an involucre and single flowers with 5–15 sepals, as his sect. Anemonanthea DC., now regarded as sect. Anemone (Candolle 1818). He subdivided the section into an unnamed group with sessile involucres and tuberous roots, in which he included *A. coronaria*, *A. pusilla* DC. non Gaertn., *A. palmata*, *A. pavonina* and *A. stellata* along with another five globally distributed species (Candolle 1818). Candolle included *A. hortensis* incorrectly as a synonym of *A. stellata*, which he stated had 10–12 oblong, more or less obtuse sepals. He described his concept of *A. pavonina* as having 10–12 acute, lanceolate sepals, adding that he had rarely seen single-flowered plants, mostly those in gardens with double flowers (see comments on *A. pavonina* under Lamarck below).

Candolle continued with his treatment of Anemone sect. Anemonanthea in the first volume of his *Prodromus* (Candolle 1824). Under his unnamed grouping of species with sessile involucres and tuberous roots, he maintained *A. coronaria* and *A. pusilla* as he had previously (Candolle 1824). He added two new varieties: A. palmata var. florepleno DC. and A. pavonina var. fulgens DC. (see full discussion on this below). Candolle once again also included *A. hortensis* as a synonym of *A. stellata* and added the significant statement pertaining to this (translated from Latin): “It is more rarely cultivated in gardens and is more often provided with a single flower than the *Anemone pavonina*, with which it has long been confused” (Candolle 1824).

The German botanist and physician Kurt Polycarp Joachim Sprengel (1766–1833), in his treatment of the genus *Anemone* a year later, divided it into four unranked subdivisions (Sprengel 1825). His second division, *Anemone* [unranked] *Stephanomata* Spreng., was described as having divided leaves, solitary flowers and villose fruits (Sprengel 1825). He further subdivided this unranked group into those plants with tuberous roots (Group 1), as opposed to those with roots that were either woody (Group 2) or fibrous (Group 3). In the former group he included *A. coronaria*, *A. formosa* Clark (see synonymy below), *A. palmata*, *A. pavonina* and *A. stellata* and another six species from various parts of the world (Sprengel 1825). He followed Candolle by incorrectly including *A. hortensis* as a synonym of *A. stellata* and distinguished *A. pavonina* from *A. stellata* and *A. formosa* by having lanceolate, acute sepals, as opposed to sepals with apices somewhat blunt in *A. stellata* and sepals broadly ovate in *A. formosa* (Sprengel 1825).

In his *Anemonarum revisio*, the German botanist Georg August Pritzel (1815–1874) included *A. coronaria*, *A. formosa*, *A. fulgens*, *A. hortensis* and *A. palmata* in an unnamed subsection characterised by woolly fruits, tuberous roots and sessile involucral leaves, within Anemone sect. Anemonanthea DC. (Pritzel 1841). His description of *A. fulgens* included sepals 8–15, oblong with obtuse apices and included a var. β in polynomial form, comprising numerous extremely narrow sepals with most acute apices, and cited for that variety *A. pavonina* Lam. in synonymy (Pritzel 1841). Pritzel described *A. hortensis* as having sepals 8–14, more or less broadly oblong-lanceolate and with somewhat obtuse apices. He also included *A. stellata* Lam. in synonymy (Pritzel 1841). Pritzel referred to Sprengel (1825) for his description of *A. formosa* as having larger, broadly ovate sepals (Pritzel 1841).

Oskar Eberhard Ulbrich (1879–1952), in his revision of *Anemone*, placed what he considered to be *Anemone pavonina* next to the Spanish species *A. palmata* in Anemone sect. Eriocephalus Ulbr. subsect. Longistylae Ulbr. series *Oriba* (Adans.) Ulbr. (Ulbrich 1905). He also included *A. hortensis* and *A. coronaria* in series *Oriba* and considered *A. fulgens* to be synonymous with *A. pavonina* (Ulbrich 1905: 202, 247). He did not provide any diagnostic characters in order to distinguish *A. pavonina* from its closest relatives, merely stating that it was a very variable species that occurs from southern France to Greece and Turkey (Ulbrich 1905). Ulbrich regarded A. pavonina var. regina (Risso) Rouy et Fouc. (albeit an invalid name; see discussion on Rouy and Foucaud under Synonymy below) as being found only in the western part of Europe, limited to southern France, northern Italy and Corsica, but again provided no morphologically diagnostic evidence for this statement. He likewise referred A. pavonina var. purpureo-violacea (Boiss.) Halacsy to eastern Europe, with a distribution from Thrace and Macedonia through Greece, including the islands of Melos, Chios and Kos, but again without providing any distinguishable characters for doing so (Ulbrich 1905).

The Italian botanist Gina Luzzatto (1904–c. 1987) undertook a very comprehensive morphological examination of *A. fulgens*, *A. hortensis*, *A. pavonina* and *A. stellata*, which included several infraspecific names. Luzzatto surveyed the literature associated with the names; however, while examining many collections in herbaria and living material from the various territories where the taxa occur, she inferred relationships based on her own observations and from the descriptions of earlier authors without reference to type material (Luzzatto 1932a, b, 1933, 1934a, b). In a field study of the area around Nice, where Luzzatto examined material of what she referred to as Anemone hortensis L. var. pavonina Lam., near Levens, she stated that the plants agreed with Lamarck’s description “in cui il fiore è spesso doppio, con un gran numero di sepali lanceolato-lineari acuti, d’un rosso scarlatto, salvo gli esterni che sono verdi” [in which the flower is often double, with a large number of acute lanceolate-linear sepals, of a scarlet red, except for the external ones which are green] (Luzzatto 1932b). Luzzatto maintained her concept of Lamarck’s double-flowered plants in her later treatment, in which she described *A. pavonina* as having (translated from Italian) “double flowers with many oblong narrow pointed sepals” (Luzzatto 1934a).

The horticulturalist Edward Augustus Bowles (1865–1954) wrote a historical assessment of what he considered to be *A. hortensis* and *A. pavonina* but did not mention Lamarck’s type material (Bowles 1948). A bibliographical summary in Bowles’s account by the botanist William Thomas Stearn (1911–2001), who was then librarian at the Royal Horticultural Society, did, however, include the statement: “the type of *A. pavonina* is the monstrous double form listed below as var. pavonina” (Stearn in Bowles 1948). It is in that paper where the first mention of Candolle’s A. pavonina var. fulgens is considered to be of hybrid origin as *A. × fulgens*. Bowles asserted that this was between what was stated to be the native *A. hortensis* and the introduced *A. pavonina*, which he concluded must have been introduced from Greece, Macedonia and western Asia (Bowles 1948).

A more recent revision of the genus *Anemone* based on examination of comparative morphology (Ziman et al. 2008) concluded that Anemone subgen. Anemone sect. Anemone subsect. Anemone comprised the species *A. coronaria*, *A. hortensis* and *A. palmata*, while subsect. Somaliense Ziman, Bulakh and Kadota comprised the single species *A. somaliensis* Hepper (Ziman et al. 2008). The three species in subsect. Anemone are distinguished from *A. somaliensis* by the basal parts of the leaf petioles having an expanded sheath and deciduous tepals, vs no basally inflated leaf sheath and persistent tepals (Ziman et al. 2008). Moreover, *A. somaliensis* differs from *A. hortensis* by having much larger involucral leaves (similar to the basal leaves) and a smaller perianth (Ehrendorfer et al. 2009). Neither *A. fulgens* nor *A. pavonina* was mentioned in the treatment by Ziman et al. (2008).

### ﻿Molecular and cytological review

Maïa and Venard (1976) undertook cytotaxonomic and phytochemical studies on *A. coronaria*, *A. hortensis*, *A. palmata*, *A. pavonina* (from southern France) and *A. variata* Jord. (see synonymy below). These authors followed the assumption, instigated by Bowles (1948), that *A. hortensis* is restricted in its origin to southwest Europe, occurring in Albania, Italy and France, whereas *A. pavonina* occurs in Greece and southwest Asia (Maïa and Venard 1976). Their morphological diagnosis of “*A. hortensis* (= *A. stellata* Lamarck)” was that it had 12 to 18 sepals, whereas *A. pavonina* (including the statement “with four varieties: typica, ocellata, purpureo-violacea and pavonina”) had nine sepals. They also followed Bowles’s classification, stating that *A. × fulgens* was a hybrid between *A. hortensis* and *A. pavonina*, adding that A. pavonina var. ocellata, with large red tepals exhibiting a basal zone of white, had been introduced into southern France from Greece (Maïa and Venard 1976).

Two samples of what they considered to be *A. pavonina* showed different chromosome lengths. One collection (sample a) was from a population of A. pavonina var. ocellata gathered near the village of Valbonne near Grasse in Provence, and the other (sample b) was from a mixed horticultural strain cultivated in the Station d’Amélioration des Plantes at Fréjus, Var, also in Provence. This difference in chromosome lengths they regarded as intraspecific variation, i.e. variation within the same species (Maïa and Venard 1976).

Their isolation of the floral pigments in *A. hortensis*, A. pavonina var. ocellata and A. pavonina var. purpureo-violacea revealed that all three samples they examined contained the pigments kaempferol (yellow) and quercitol (off-white), and that both their samples of *A. hortensis* and A. pavonina var. purpureo-violacea contained cyanidol (or cyanidin, blue or red), which was lacking in A. pavonina var. ocellata, where it was substituted by pelargonidol (or pelargonidin, orange, red or red-blue). The floral pigments, however, in *A. coronaria* included all five pigments (Maïa and Venard 1976).

Maïa and Venard concluded that the chromosome morphology of *A. palmata* was very different from the group comprising *A. coronaria*, *A. hortensis* and the two samples of *A. pavonina*. They stated that the chromosomes from *A. hortensis* and sample (a) of A. pavonina var. ocellata did not differ from each other and that the chromosome lengths of *A. hortensis* and both samples of *A. pavonina* also coincided in length. They also ascertained that within the second group, the chromosome morphology of *A. coronaria* was quite distinct from the other three samples (Maïa and Venard 1976).

Hoot et al. (1994) undertook phylogenetic analyses based on combined data from cpDNA restriction sites, ribosomal DNA restriction sites and morphology but did not include data for *A. palmata*. Their results confirmed the sister relationship between *A. coronaria* with pantoporate pollen and the spiraperturate pollen of *A. fulgens* sensu Hoot et al., *A. hortensis* and *A. pavonina* sensu Hoot et al. (Hoot et al. 1994). Their results, obtained from the phylogenetic analyses of restriction site data, showed that a clade with *A. coronaria* on a branch with 13 sites was sister to a polytomy comprising *A. hortensis*, *A. fulgens* and *A. pavonina*, each with zero restriction site variation (Hoot et al. 1994). The phylogenetic tree from the combined analysis of morphological data, cpDNA and restriction site variation revealed the same topology for the four sampled taxa, sister to a clade with four species occurring in the southwest USA. The clade with *A. coronaria* sister to a branch with *A. hortensis*, *A. fulgens* and *A. pavonina* was referred to as part of the “Coronaria Group” within Anemone sect. Anemone (Hoot et al. 1994).

Ehrendorfer and Samuel (2001) sequenced the plastid *atpB/rbcL* intergenic spacers of 21 taxa of *Anemone*, obtaining a single most parsimonious tree. They included samples of *A. coronaria*, *A. hortensis*, *A. palmata* and a Greek sample from Olympia, Peloponnese, of their understanding of *A. pavonina* (Ehrendorfer and Samuel 2001). Their results showed moderate support (68% bootstrap) for what they termed the “Coronaria” clade, comprising *A. coronaria* and two samples of *A. palmata* sister to *A. hortensis* and *A. pavonina* on a branch with 85% bootstrap support (Ehrendorfer and Samuel 2001). In their discussion they concluded that “*A. hortensis* and *A. pavonina* are close and possibly only elements of one polymorphic Mediterranean species, whereas the frequently sympatric *A. coronaria* appears well separated” (Ehrendorfer and Samuel 2001).

The results from the study by Ehrendorfer and Samuel (2001) were corroborated by results from cytogenetic data by Mlinarec et al. (2006) from Adriatic populations of *A. hortensis*, including samples of what they referred to as *A. pavonina*. Their results revealed that *A. coronaria*, *A. hortensis* and *A. pavonina* all had three acrocentric and five sub-metacentric chromosomes and all had similar heterochromatin banding patterns (Mlinarec et al. 2006). They concurred with the results from Hoot et al. (1994) and Ehrendorfer and Samuel (2001), stating that “*A. hortensis* and *A. pavonina* are the most closely related species within the Coronaria group” (Mlinarec et al. 2006).

The revised molecular and morphological study undertaken by Ehrendorfer et al. (2009) has enormously clarified the species relationships and ascertained the wild distribution of the taxa in Anemone sect. Anemone. It has also gone a long way towards resolving the extremely muddled taxonomic history of both *A. coronaria* and *A. hortensis* (Ehrendorfer et al. 2009). The classification in the study was based on sequence data from the cpDNA intergenic region *atpB-rbcL* previously undertaken by Ehrendorfer and Samuel (2001). This study has broadly expanded on the phylogenetic analyses of Hoot et al. (1994); however, these authors admitted that the variation within *A. hortensis* had not been sufficiently studied. They treated material classified as *A. fulgens*, *A. pavonina* and *A. stellata*, and other infraspecific named taxa, as variations within a polymorphic *A. hortensis* (Ehrendorfer et al. 2009).

The topologies for these taxa in the studies by Hoot et al. (1994) and Ehrendorfer and Samuel (2001) are broadly congruent with the results from analyses of chloroplast *atpB-rbcL* spacer and nuclear ITS regions undertaken on 55 species of *Anemone* by Hoot et al. (2012). The results by Hoot et al. (2012) indicated a Bayesian probability value (BI) of 1 and 82% maximum parsimony bootstrap support for the clade comprising *A. coronaria*, *A. somaliensis*, *A. hortensis* and *A. pavonina* (Hoot et al. 2012). One branch on this clade comprised *A. coronaria* and *A. somaliensis* (BI 1; 78% bootstrap), sister to a branch with *A. hortensis* and *A. pavonina* (BI 1; 100% bootstrap). The study by Hoot et al. (2012) recognised *A. coronaria*, *A. hortensis*, *A. palmata*, *A. pavonina* and *A. somaliensis* as forming Anemone subgen. Anemone sect. Anemone subsect. Anemone ser. Anemone (Hoot et al. 2012). These authors stated that although *A. hortensis* and their understanding of *A. pavonina* sampled from Greece are very well supported as sisters to each other, the branch lengths indicated a number of unique substitutions, suggesting that species status might be correct for *A. pavonina*, but they did not include any indications as to potential differences in morphology (Hoot et al. 2012).

Examination of relict satellite DNA sequences of the AhTR1 group in 22 species from four sections of *Anemone* also showed that *A. hortensis* from the island of Hvar, Croatia, and their sampling of *A. pavonina* from Bogdanci, Paljurci, North Macedonia, shared a very similar species-specific satDNA profile. Their results support the treatment by Ehrendorfer et al. (2009) that *A. hortensis* and *A. pavonina* are a single species (Besendorfer and Mlinarec 2013).

The topologies from all previous molecular analyses are congruent with results from sequence data from the nuclear 5S rDNA gene and spacer region (Mlinarec et al. 2016). The latter’s research showed that four samples of *A. hortensis* from Croatia and four samples of their understanding of *A. pavonina* from North Macedonia formed a clade with Bayesian inference of 1 but were unresolved with respect to a strongly supported clade of three samples of *A. palmata*, also with BI of 1, and a clade of eight samples of *A. coronaria*, also with BI of 1. These taxa formed a discrete clade with moderate support of 0.62 BI, which was referred to incorrectly as “sect. Coronaria” (Mlinarec et al. 2016). Branch lengths on the phylogram for the samples on each of the three branches on the clade representing *A. hortensis* and *A. pavonina*, *A. palmata* and *A. coronaria* all showed differing lengths. The lengths shown by the three samples of *A. pavonina* and four samples of *A. hortensis*, albeit well supported, all showed greater differences in their branch lengths than did the eight samples of *A. coronaria* (Mlinarec et al. 2016). Branch lengths between the three samples of *A. pavonina* and four of *A. hortensis* were, however, comparable to the differences in branch lengths of most other species of *Anemone* in that study.

The most recent phylogenetic study using data from cpDNA (*atpB-rbcL*, *trnL-F*, *rbcL*) and nrDNA ITS sequences on 159 individuals representing 146 species of *Anemone* was conducted using maximum likelihood (ML) and Bayesian inference (BI) (Xiang et al. 2024). Their results also concurred with the topologies of those from previous molecular analyses. A large clade of 50 species was fully supported (100% bootstrap, BI 1), which was referred to as sect. Anemone (Xiang et al. 2024). Within that large clade, *A. coronaria*, *A. hortensis*, *A. palmata*, *A. pavonina* and *A. somaliensis* all formed a moderately supported clade (58% bootstrap; BI 1) comprising two subclades: *A. palmata*, *A. hortensis* and *A. pavonina* sister to *A. coronaria* and *A. somaliensis* (Xiang et al. 2024). The subclade with *A. palmata* sister to *A. hortensis* and *A. pavonina* was also moderately supported (67% bootstrap, BI 1), while the branch with *A. hortensis* and *A. pavonina* was well supported (83% bootstrap, BI 1).

### ﻿*Anemone hortensis* L.

Linnaeus validated *A. hortensis* in his “Species plantarum” with the following protologue:

*Anemone hortensis* L. Sp. pl. 1: 540 (1753)

9. *Anemone foliis* digitatis

*Pulsatilla foliis* digitatis Hort. cliff. 224.

*Anemone geranii* rotundo folio, purpurascens. Bauh. pin. 173.

*Anemone hortensis latifolia* 3. Clus. hist. 1. p. 249.

*Anemone* 1 Dod. pempt. 434. (in fact 430/431)

Habitat ad Rhenum & in Italia ♃ (= perennial sign)

Due to their apparent morphological similarity, Linnaeus was initially uncertain as to how he could distinguish between *Anemone coronaria* and *A. hortensis*. He described both species under the group heading of “Anemones caule folioso, seminibus caudatis” [Anemones with stems bearing leaves and seeds with tails] (Linnaeus 1753). His validating description of *A. coronaria* was “7. Anemone foliis radicalibus ternato-decompositis, involucro folioso” [*Anemone* with much-divided ternate basal leaves, involucre leaf-like]. He also included a variety of *A. coronaria* with red flowers and multiple tepals, thereby recognising that *A. coronaria* with its narrow leaves also exhibited doubling of the floral parts “β Anemone tenuifolia multiplex rubra”. He stated that the origin for both varieties of *A. coronaria* was from “Oriente, Constantinople allata”, i.e. brought to me from Mediterranean eastern Europe and Constantinople (Linnaeus 1753).

Prior to the publication of his protologue, Linnaeus had alluded to the variation in colour and the number of tepals of *A. hortensis* “variat florum plenitudine et colore” on what he had then called “Pulsatilla foliis digitatis” (Linnaeus 1738 see Reference 1 below). His initial distinction between *A. coronaria* and *A. hortensis* was, however, largely based on leaf dissection – the former had much more dissected ternate leaves, whereas the leaves of *A. hortensis* were digitate. He indicated that *A. hortensis* was to be found in Germany along the river Rhine and in Italy (see References 1 and 3 below).

Linnaeus maintained his brief description for *A. hortensis* in the second volume of the tenth edition of “Systema naturae” (Linnaeus 1759). In that work he did not provide any additional information for the species, maintaining the simple phrase which he had used in his protologue, “9. *A. foliis* digitatis [*Anemone* with digitate leaves]” (Linnaeus 1759). The description for *A. hortensis* was repeated verbatim in “Species plantarum” ed. 2, but for *A. coronaria*, which again included his β variety, he added another variety, “γ *Anemone angustifolia* multiplex, mutata florum facie quotannis nova” [*Anemone* with multiple narrow leaves and flowers which alter their appearance every year] (Linnaeus 1762).

In the “Systema naturae” ed. 12 vol. 2, the entry for *A. coronaria* is the same as that in “Systema naturae” ed. 10, but for *Anemone hortensis*, it is enlarged to “9. *A. foliis* digitatis. *Anemone* fol. digitatis: lobis incisis, petalis lanceolatis numerosis. Ger. prov. 380 [*Anemone* with digitate leaves with incised lobes, petals lanceolate, numerous]” (Linnaeus 1767). The reference to “Ger. prov.” is to the French physician Louis Gérard (1733–1819), who wrote “Flora gallo-provincialis” (Gérard 1761). Gérard’s description of the anemone follows below.

The description of *A. hortensis* in the 12^th^ edition is repeated verbatim in “Systema naturae” ed. “13” (Linnaeus 1770) and again in the “Systema vegetabilium”, with the addition only of “seminibus lanatis” [woolly seeds] (Linnaeus 1774).

Linnaeus’s most comprehensive description of *Anemone hortensis*, however, was in his *Mantissa altera* 2, which was in effect an appendix to the 12^th^ edition of “Systema naturae”: “*Anemone hortensis*. Involucrum foliolis lanceolatis. Petala 9, lineata, extus pubescentia” [*Anemone hortensis*. Involucre of lanceolate leaflets. With 9 petals, each with lines and pubescent on the outside] (Linnaeus 1771).

Louis Gérard, who was cited in “Systema naturae” ed. 12, used the phrase name “5. *Anemone foliis* digitatis, lobis incisis, petalis lanceolatis numerosis” (Gérard 1761). Gérard added the following synonyms:

*Anemone foliis* digitatis. Linn. Spec. 540.

*Anemone geranii* rotundo folio, purpurascens. C. B. Pin. 173. Tour. 176.

*Anemone italica*, latiusculis spinosis foliis, tertia clusii. J. B. 3. p. 402

*Anemone* 1. Dod. pempt. 434. Dalech. p. 845.

*Anemone* 2. Camer. epit.

“Provenit in sterilibus abbatiae” du Thoronet. Perennis.

Gérard described this species from waste ground around the Abbey of Thoronet, c. 20 km from his home in Cotignac, Provence, and referred to Linnaeus’s protologue but without mentioning the name *A. hortensis*. Gérard referred to an illustration in Daléchamps (1587). This illustration was the same, reversed, as that already used in Clusius (1576) and in L’Obel (1576, 1581) and again in Dodoens (1583). Gérard included a reference to Tournefort (1700), who, in turn, cited Caspar Bauhin (C. Bauhin 1623). Gérard also referred to an engraving in Camerarius (1586). All the illustrations cited by Gérard depict *A. hortensis* L.

#### ﻿Linnaeus’s references

His first reference was to his own work: “4. *Pulsatilla foliis* digitatis” in his “Hortus Cliffortianus” (Linnaeus 1738). Between 1735 and 1738, Linnaeus catalogued the plants in the de Hartekamp garden near Haarlem in the Netherlands, belonging to the rich Dutch East India Company director George Clifford III.

Linnaeus added additional references in the catalogue to the following sources, either directly or indirectly: L’Obel (1576), Dodoens (1583), Daléchamps (1587), Clusius (1601), C. Bauhin (1623), J. Bauhin et al. (1651), Morison (1680), Boerhaave (1727).

Linnaeus’s description in the Hortus Cliffortianus (Linnaeus 1738) included habit and locality information: “Crescit in vepretes in Germaniae quibusdam locis secundum Rhenum, inter Moguntiam et Andernacum; in collibus asperis apricis maritimis prope Pisas in monte San Juliani; in montibus Bononiae vicinis, inter Lericam et Massam Liguriae urbem copiosa; in multis locis Italiae [It grows in thickets in certain parts of Germany along the Rhine, between Mainz and Andernach; in the rugged and sunny maritime hills on Monte San Giuliano near Pisa; in the mountains around Bologna, abundantly between the Ligurian towns of Lerici and Massa; in many parts of Italy]”.

Variat florum plenitudine et colore [It varies in the fullness and the colour of the flowers].

Folia ad petiolum usque multifida sunt, et respectu periphaeriae peltata” [the leaves are peltate, divided into several parts from the petiole].

Linnaeus’s habitat information, in which he stated that the species occurs along the river Rhine, is almost certainly to have been taken from the account provided by Clusius (Clusius 1576; 1601).

Linnaeus’s second reference was “Anemone geranii rotundo folio, purpurascens” in Caspar Bauhin’s *Pinax*. Bauhin included this as his second species entry under the general heading “Anemone latifolia simplici flore” [anemone with broad leaves and simple flowers] with an additional ten references (C. Bauhin 1623). Bauhin’s description, lacking any accompanying illustration, was clearly influenced by that of Dodoens (1583). Bauhin stated (translated from Latin): “The flower is made up of 12 or 15 leaves [i.e. tepals], greyish hairy outside, shining purplish-red on the inside: thence to a lighter reddish-purple: sometimes the inside is more intense, the outside is more soft: rarely is it completely white” (C. Bauhin 1623). His additional references included flower colours of both crimson (kermesina) and purple (purpuro saturo).

Linnaeus included as his third reference Carolus Clusius’s “Anemone hortensis latifolia 3” (Clusius 1601). Clusius included under the heading “Anemone hortensis simplici flore Cap. LVII” a clearly identifiable illustration of *A. hortensis* as “Anemone hortens. latif. simpl. flore iii” and mentioned in his description that the leaves were like those of *Sanicula* [i.e. *S. europaea* L.] (Apiaceae). Clusius’s description was clearly the basis for Linnaeus’s own description in *Hortus Cliffortianus* (Linnaeus 1738). Clusius stated (translated from Latin): “Origin: In some parts of Germany, along the fertile Rhine, between Mainz and Andernach, I saw it blooming among the scrub in the sun in March. Even after the year 1580 (for I do not remember seeing the mass of it before then), it was brought to Constantinople, or at least that was a plant very similar to it, if not the same. Cultivated in gardens, it produces most of its leaves before the winter, but it flowers in the spring and sometimes even in the autumn: the seed ripens in May, which is immediately given up to the wild, as I have often experienced. I think that it was a native in the same way in Italy, because a few years ago I received it from the learned man Ferdinando Imperato of Naples” (Clusius 1601).

Clusius had in fact previously mentioned the plant that he called “Anemone latifoliaaltera number 2” as occurring near the river Rhine (Clusius 1576). His text read (translated from Latin): “I have seen it in March blooming in the sun in some parts of Germany along the Rhine. With us it is cultivated in gardens and comes out before winter, but it blooms in spring, sometimes even in autumn: the seed ripens in May” (Clusius 1576). This text is accompanied by an illustration (Clusius 1576) which he later reused (Clusius 1601).

Clusius stated that he had received the plant from the Neapolitan herbalist and apothecary Ferdinando [Ferrante] Imperato (1525–1621), who published “Dell’ Historia Naturale” in 1599 and who had established a Museum of Curiosities in Naples (Clusius 1601). Imperato’s description and illustration (Imperato 1599), however, appear to represent either *Adonis annua* L. or *Anemone coronaria*. In short, Imperato’s entry appears not to be connected with the specimen discussed by Clusius.

Clusius’s reference to the plant being brought to Constantinople “or at least a plant very similar to it” was in fact a reference to *Anemone coronaria*, as he explained later in his “Atrebatis rariorum aliquot stirpium: per Pannoniam, Austriam” (Clusius 1583). He said, when referring to number 1 “Anem. tenuifol. coccineo flore” and number 2 “Anem. tenuifol. carneo flore”, but without illustrations (translated from Latin): “All these do flower in April and are without odour: I first saw them blooming in the cultivated garden of the great lady de Heusenstain in the years 1582 and 1583, not without pleasure” (Clusius 1583). His Viennese gardening friend Anna-Maria von Heusenstain Starhemberg, whose husband was an influential statesman in the court of Emperor Maximilian II, whom Clusius had befriended during his time in Vienna from 1573 to 1588, corresponded with him until 1606, providing him with many plants that she had been sent from her contacts in Constantinople (Egmond 2010; 2013).

It may, however, have been Anna-Maria von Heusenstain who had indeed given Clusius *Anemone hortensis* under the name “Anemone latifolia Byzantina simp. flo.” (Clusius 1583). He stated, “Furthermore, another broad-leaved Anemone, which was brought to me from Byzantium in the autumn of 1581, at first, when I saw the leaves emerging from the ground, I thought it was similar to that which is cultivated in Belgian gardens with a large purple flower consisting of 12 or more leaves [petals]. But the flower subsequently unfolding itself with the others in April and this year 1583 taught me that it was different, and that two different kinds of it were found” (Clusius 1583).

Linnaeus’s fourth and last reference was to Rembert Dodoens’s number 1 of the “De Anemonibus” in his “Stirpium historia pemptades sex” (Dodoens 1583). Dodoens described his Anemone 1 with: “Color flori elegans est, in dilutiori purpura rubens; qui quandoque ab utraque, alias ab interiore tantum relucet parte intensior, exterius remissior et inalbicans. Huius generis et albida sunt flores, sed rariores” [The colour of the flower is elegant, in a pale purplish-red, which sometimes shines from both sides; at other times more intensely only from the inner part, the outer part is softer and whiter. There are also white flowers of this kind, but rarer” (Dodoens 1583). There is an illustration representing *A. hortensis* with several incised leaves and four flowers, one in bud, another in seed and the remaining two each with ten to twelve narrow tepals (Dodoens 1583). Dodoens does not provide any information as to the provenance of this species.

#### ﻿Linnaeus’s original material

There are four sheets of original material for this name, two in the Clifford Herbarium (BM) and two in the Linnaean Herbarium (LINN). The lectotype (Fig. 1C), designated by Arne Strid in Jarvis et al. (2005: 469), is sheet 224 cited in the protologue [BM000628826], gathered from the garden of George Clifford III in the Netherlands (Linnaeus 1738). The sheet has three palmatifid, lobed leaves and two flowers, one with ten and the other with twelve obovate tepals. There is a strong residual reddish-pink tinge to the two flowers, and each acute or obtuse tepal has five main longitudinal veins. The two flowering stems have an involucre of three lanceolate leaflets below each flower; one leaflet on the right-hand flower stem is dissected. The second sheet in Clifford’s Herbarium [BM000576288] has two annotations written by an unknown hand (C. Jarvis, pers. comm.): “*Anemone foliis* digitatis Linn. Sp. pl. 2. p. 761. n. 9.” and “*Anemone geranii* rotundo folio, purpurascens. CBp. 173. M.H.2. 425. *A. hortensis*latifolia simplici flore Clus. H. 249. *A. italica* latiusculis spinosis 3. Clusii JB.3. 402.” The last reference is Johann Bauhin’s reference to Clusius’s phrase name, *Anemone italica* latiusculis spinosis foliis number 3, which has an accompanying illustration (J. Bauhin et al. 1651).

On this sheet are four lobed and palmatifid leaves, each varying from the others in the amount of lobing and dissection. These leaves might have originated from different plants or from different stages of growth on one plant. The two flowers on the sheet sit above an involucre of undivided, lanceolate leaflets and have ten and eleven obovate tepals that are broader than those on the lectotype sheet. The tepals on one flower have a faint pinkish tinge and five longitudinal veins.

There are also two sheets in the Linnaean Herbarium (LINN). The first sheet is Herb. LINN 710.15. It has written on it “hortensis” and immediately beneath the plant “Capell” and on the reverse of the sheet “In sylvis Romae”. The annotation “Capell” on this specimen is likely to refer to Moritz Anton Cappeller (1685–1769), a Swiss physician and naturalist born in Willisau who practised in Lucerne, c. 32 km (20 miles) to the south-east of Willisau. During the War of the Spanish Succession between 1701 and 1714, he served as a doctor and engineer in Naples. It seems possible that he may have collected the specimen in woods near Rome on his way back to Lucerne. Communication of the specimen to Linnaeus may have been via a third party such as the Zurich physicians Johannes von Muralt (1645–1733) or his student Johannes Gessner (1709–1790) or the botanist and anatomist Albrecht von Haller (1708–1777), as they all shared a mutual interest in natural history. Cappeller, Gessner and Linnaeus also shared a specific interest in mineral crystals, although no correspondence between them to that effect has been found (Jarvis 2007). The sheet LINN 710.15 has one whole plant on it and, in addition, two flowering stems from other plants. The leaves are more divided on the single plant, and the sixteen tepals on the stem are narrower than on those of the Clifford specimens, each tepal with three longitudinal veins. The two single flowering stems have tepals that are also lanceolate rather than obovate compared to those on the Clifford sheets, and both have thirteen tepals with three veins.

The second sheet, Herb. LINN 710.14, has written on it “hortensis” and, later, in James Edward Smith’s hand, “stellata De Cand 14”. This also comprises a single plant with dissected leaves, as in LINN 710.15, and fifteen narrow tepals, each with three veins.

There is a sheet of a specimen of *A. hortensis* with eleven lanceolate, three-veined tepals in Linnaeus’s Herbarium (S-LINN) in Stockholm sent by Andreas Dahl (1751–1789) to Linnaeus annotated “Anemone hortensis Dahl a Linné S” without additional data [S09-28144].

### ﻿*Anemone pavonina* Lam.

Jean-Baptiste-Pierre-Antoine de Monet, chevalier de Lamarck (1744–1829), described *Anemone pavonina* in his “Encyclopédie méthodique”. Botanique 1(1): 166 (1783). His protologue was:

11. Anémone oeil de Paon, *Anemone pavonina*.

*Anemone foliis* radicalibus profunde tripartitis, lobis cuneatis, incisis, dentatis; flore variegato [Anemone with basal leaves deeply divided into three parts, lobes wedge-shaped, incised and toothed, flowers variegated].

*Anemone latifolia, pavo dicta major* [broad-leaved anemone, called the greater Pavo]. Bauh. Pin. 176. No. 4, 5, 6. *Anemone latifolia* maxima versicolour [Large broad-leaved anemone with variously coloured flowers]. Bauh. Pin. 176. Tournef. 276.

A full description followed (translation from French): “This species, which has been cultivated for several years in the Jardin du Roi, produces flowers of a very pleasant appearance, of a form quite different from that of the *Anémone* des Fleuristes [i.e. his number 9. *A. coronaria*], and which blooms from the beginning of April. Its root is thick, tuberous, furnished with lateral fibres, and bears leaves which closely resemble those of the Sanicle officinale [*Sanicula europaea* L.]. These leaves are petiolate, deeply divided into three widened, wedge-shaped lobes, unequally incised, and ending in coarse teeth whose tips point in different directions. The stem is seven or eight inches high, a little hairy, and provided at two-thirds of its height with a small collar of three smallish leaves, two of which are very often simple, and the third a little divided. At the top of this stem is born a flower coloured with red and white, almost an inch and a half wide, composed of many oblong, slightly narrow, pointed petals, of which the interior ones are the smallest. These petals are veined longitudinally, slightly hairy on their reverses, whitish at their base, and a beautiful red towards their apex, and they have this remarkable feature that the exteriors are scarcely coloured, sometimes even entirely green, so that they appear to form a calyx contiguous to the corolla, as in the preceding species [i.e. his number 10. *A. palmata*]. It is evident, however, that these two plants have no other natural calyx except the small collar itself, which they carry a little below their flower: this part corresponds entirely to the small calyx of the hepatic *Anémone* [*A. hepatica* L. = *Hepatica nobilis* Schreb.], which is also a little distant from the corolla, a characteristic common to all the species of this genus.

The species I have just mentioned is, I believe, native to the Levant; and although I have only seen it with double flowers, there is no doubt that the natural plant which is typical of this species is very different from that which constitutes the *Anémone* des Fleuristes, n°. 9. It is cultivated in the gardens of the Curious: there it produces agreeable varieties (v. v.)”.

Lamarck initially recognised the existence of *Anemone hortensis* in the third volume of his “Flore Françoise” (Lamarck 1779), citing Linnaeus’s *A. hortensis* from the second edition of “Species plantarum” (Linnaeus 1762). Lamarck diagnosed his species number 18, Anémone des jardins, *Anemone hortensis*, with the sentence “Plus de sept petales, semences simplement laineuses [more than seven petals, seeds with simple woolly hairs]. He made no reference in this work to *A. coronaria*. He described the three-lobed digitate leaves, the collarette or involucre comprising three sessile, more or less incised leaflets, and the single flower composed of nine lightly purplish, narrow and veined petals which are a little velvety on their underside. He added that it grew in waste ground in Provence and that there were bright-coloured varieties of it grown in gardens (Lamarck 1779). Lamarck’s only other reference was Tournefort’s *Anemone geranii* rotundo folio, purpurascens, already cited above under Gérard (Tournefort 1700).

Four years later, in the first volume of his great work, the “Encyclopédie methodique. Botanique,” Lamarck placed both *Anemone coronaria* and his two new species, *A. pavonina* Lam. and *A. stellata* Lam., in the group of species he identified as having “semences chargees de duvet, mais non munies de longues queues plumeuses” [seeds covered with down but not provided with long feathery tails] (Lamarck 1783: 165). He included under *A. coronaria* two synonyms without additional descriptions: (*a*) *Anemone hortensis*, *tenuifolia* and (*β*) *Anemone hortensis*, *latifolia*. Following his description of *A. coronaria*, he also described two new species: *Anemone pavonina* and *A. stellata* (Lamarck 1783: 166).

Lamarck named the species the ‘eye of Paon’. According to Clusius, this was in reference to the orbs or eyes seen at the ends of the peacock’s tail feathers and was probably an allusion to the presence of a circle of gold or white formed from the pale bases of each of the tepals on some plants of the species. These paler zones surrounding the darker centre of the flowers of “Anemone hortensis latifolia pavo major 1” act as the eye’s pupil (Clusius 1601: 261). In Clusius’s own description of the flowers of “Anem. hort. latifol. pleno flore 1”, he stated (translated from Latin): “then emerging two inches above the aforementioned leaves [involucre], it supported a large and widely spread flower, consisting of numerous leaves [tepals], the outer and larger ten or twelve of which were mostly green in colour, their tips lightly and linearly stained with a scarlet colour, while the inner ones, smaller and narrower, shone with a more diluted red-purple, and those covering the middle flower were also of the same colour, reflexed to the navel, and as if clustered” (Clusius 1601: 261).

The Latin generic name *Pavo* L. is the name for peafowl (Linnaeus 1758). The famous “eyes” found on the tail feathers of the male peacock probably appertain to the legend of Zeus and his flirtation with Io, a mortal lover of Zeus, who was changed by him into a heifer in order to be concealed from his wife, Hera. Suspicious Hera consequently sent the all-seeing beast Argos Panoptes (or Argus) with his multitude of eyes to keep watch over Io. Gaia, goddess of the earth, had sympathy for Io and provided the red, purple and white flowers of the violet (Io’s flower) to the heifer as food. Zeus, angry with Hera for sending Argos to watch over Io, then sent Hermes to kill Argos, an act which inevitably enraged Hera, who consequently sent a stinging insect to pursue the escaped Io over land and sea (Ionian Sea). Io crossed the Bosporus (Oxen crossing) and was later transformed by Zeus back into her original mortal form, while Hera preserved Argos’s eyes for eternity locked inside the tail-feathers of peacocks.

Lamarck’s distinctions between *A. coronaria* and his *A. pavonina* correctly referred to the more finely divided and incised leaves of *A. coronaria* as opposed to the three broader wedge-shaped and lobed leaves of his *A. pavonina* having placed Linnaeus’s *A. hortensis* as a variety of *A. coronaria* as var. (*β*) *Anemone hortensis*, *latifolia*Lamarck 1783: 166). In addition, he alluded to the flowers of both the single and the double or multi-tepalled flowers of *A. coronaria*, a phenomenon which occurs in both species. In the second tome of Lamarck’s illustrated work “Tableau encyclopédique et méthodique” are illustrations by L. Fossier of both the single- and double-flowered forms of *A. coronaria* (Lamarck 1794: t. 496 figs a and b). It is of note that Lamarck did not include any illustrations of *A. hortensis* in that work. Nor was *A. hortensis* included by Poiret in the first part of the Supplement to the “Encyclopédie méthodique. Botanique,” even though the illustration of *A. coronaria* was specifically referred to (Poiret 1810). It is worthwhile noting that Poiret did not add the figure legends to Fossier’s illustrations of planche 496 in Lamarck’s original work until the publication of tome 3 of “Tableau encyclopédique et méthodique” (Poiret 1823).

Lamarck compared the rose, white, red, yellow, violet or blue coloured oval-oblong tepals of *A. coronaria* to the flowers of *A. pavoninа*, which he stated comprised many narrow tepals which are red with white at the base and which get smaller towards the centre of the flower. He indicated that he had seen living plants (v. v. = vidi vivam) and, significantly, that he had only seen plants with double flowers (Lamarck 1783).

#### ﻿Lamarck’s references

**Figure 2.**
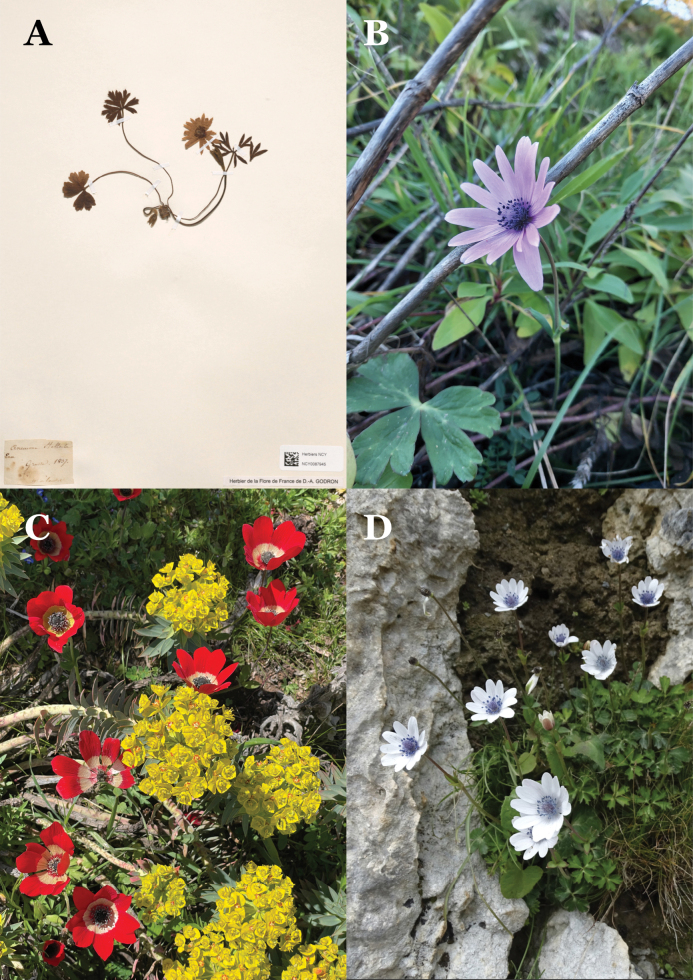
Morphological variation within *Anemone hortensis*. A. Lectotype specimen of Anemone hortensis var. stellata (NCY0087945) collected by C.J.A de Baudot near Grasse, Provence, France, in 1837, showing narrower lanceolate and more numerous tepals than in A. hortensis var. hortensis; B. Anemone hortensis var. stellata in Calabria, Italy. Photo credit: Alberto Capuano; C. Anemone hortensis f. regina (Risso) J.Compton showing basal pale yellow zonal tepal colouration, Mount Parnonas, Peloponnese, growing with *Euphorbia rigida* M.Bieb. elev. 1100m. Photo credit: Tania Compton 8 April 2024; D. Anemone hortensis var. heldreichii Crete, Mount Kouroupa, elev. 800 m, showing small white flowers with elliptic tepals Photo credit: Grzegorz Grzejszcz.

In his protologue for *A. pavonina* Lamarck’s first reference cited Casper Bauhin’s Pinax 176, which included four polynomials under the general heading of “Anemone flore pleno” [Anemone with double flowers]. Lamarck cited Bauhin’s numbers: I, IV, V and VI, all of which referred to plants with double flowers (C. Bauhin 1623). Although Bauhin (1623) did not include any illustrations to verify his descriptions, he referred under his number IV to Clusius’s “Anem. Hort. latifol. Pavo major 1, whose description of the flower is worth repeating here (translated from Latin): “then a pedicel arose from between them [the involucre], three, four, or sometimes more, inches long, thick, firm, pubescent, on which sat a single flower, consisting of twenty, thirty or more leaves [tepals] an inch or even longer, endowed with an elegant, saturated and shining scarlet colour, whose blood-red claws were surrounded by a large pale globe which added great beauty to the flower: the flower was surrounded by a black hairy head occupying the navel, surrounded by blood-red stamens with blue-green tips”. Clusius included two illustrations of different variations of the double-flowered plants (Clusius 1601). Bauhin’s number V referred to Johan de Bry’s “Anemone Pavota latifolia multiplex flore miniato” from his “Florilegium novum” (Bry 1612). Bauhin’s number VI is Clusius’s statement comparing the double with single-flowered variants. Clusius stated, but without including an illustration (translated from Latin): “The other produces flowers that are less full and sometimes consists only of a simple series of eight, nine, or ten leaves [tepals]; otherwise, it differs neither in its leaves nor in the size of the flower nor in its colour” (Clusius 1601). Bauhin, under the same number, also included a reference to Basilius Besler, whose illustration of “Anemone hortensis, flore pleno coccineo, latifolia” for the “Hortus Eystettensis” ordo 1 folio 18 fig. 2 is of a multi-tepalled plant (Besler 1613).

Lamarck’s second reference was Tournefort 276 (Tournefort 1700), which included a repeat of C. Bauhin’s “Anemone latifolia maxima versicolour” as well as his brother Johann Bauhin’s “Anemone latifolia, flore pleno variegato” (J. Bauhin et al. 1651) and also Clusius’s “Anemone hortensis flore pleno” (Clusius 1601).

Lamarck’s third and last reference was once again to Casper Bauhin, but in this case to the number 1 plant under Bauhin’s “Anemone flore pleno” grouping i.e “Anemone latifolia maxima versicolour” (C. Bauhin 1623). Under the name “Anemone latifoliae flos multiplex”, cited by Bauhin as “Anem. latifolia flore multiplici”, the multi-tepalled variant of *Anemone hortensis* was illustrated in Clusius’s “Atrebatis rariorum aliquot stirpium per Pannoniam, Austriam” (Clusius 1583). Bauhin included a third reference to Clusius’s “Anemone hortensis latifolia pleno flore 1” in Clusius’s later “Rariorum plantarum historia” (Clusius 1601). This, with its illustration entitled “Anem. hort. latifol. plen. flor. versicolour” on the following page, was in large part the basis for Lamarck’s own description of *Anemone pavonina* one hundred and eighty-two years later. Under Bauhin’s number 1 was also included “Anemone maxima polyanthos Chalcedonica” from Matthias de L’Obel’s “Icones” (L’Obel 1581). Bauhin also cited “Anemone Chalcedonica major” from the illustrations which were subsequently published as “Eichones plantarum seu stirpium” (Tabernaemontanus 1590) for Jakob Dietrich or Jacobus Theodorus (better known as Tabernaemontanus). All these references described and included illustrations of only double-flowered plants.

#### ﻿Lamarck’s original material

Two sheets have been located in the Lamarck Herbarium in P: the first [P00282069] is a specimen with two separate basal leaves and a stem with an involucre of three lanceolate leaflets and a single red flower comprising nine tepals. It has a label with “Anemone pavonina encycl” in Lamarck’s hand. Lamarck’s Herbarium, however, was sold to the University of Basel, Switzerland, in 1824, then to the University of Rostock, Germany, in 1875 and eventually to Paris in 1887. It is possible, therefore, that misrepresentations and mislabelling had occurred during these transfers. Moreover, this specimen has been remounted and may not have been the one to which the label was originally attached (Cécile Aupic pers. comm.). A label in an unknown hand has written on it, “Anemone assez rare, dans les montagnes de Nice. On la trouve double en plus grande quantité. Je n’ai pas pu scavoir son nom. [A rather rare Anemone, in the mountains of Nice. It is found double there in much greater quantity. I wasn’t able to know its name.]”. The sheet has a small red “Type” label attached by an unknown hand. Lamarck’s statement in his protologue, “quoique je ne l’aie vue qu’à fleurs doubles” or “I have only seen it with double flowers” (Lamarck 1783: 166), leaves no doubt, however, that any specimen in the Lamarck herbarium with single flowers was not seen by Lamarck when he was preparing the account of *Anemone pavonina* in the “Encyclopédie méthodique” and so would not be eligible as a lectotype. This sheet may therefore have been a later addition to Lamarck’s Herbarium and fits the circumscription of *A. hortensis* s. str.

The second sheet from Lamarck’s Herbarium (Fig. 1D) has the “Herbier de Lamarck” label but without additional annotations [P00282070]. This sheet has three separate basal leaves, involucres with five leaves per stem and a single flower on each one comprising very many narrow linear-lanceolate tepals forming two double-flowers. This specimen clearly refers to Lamarck’s description of *Anemone pavonina*. It is worth noting that although no formal typification of *A. pavonina* has hitherto been made, material matching the description was recognised to be the type of the name by Davis et al. (1984), who stated, “Described from a double-scarlet flowered specimen cultivated at Paris”.

There are two further plants on a single sheet with double-flowers in the Paris herbarium [P03182071]. The annotations on the sheet indicate that this variant was in cultivation in the Jardin des Plantes in Paris (formerly the Jardin du Roi). The sheet has two labels: on the left-hand specimen “Anemone pavonina duplex ex hortis” and on the right-hand specimen “Anem. pav. hort. Paris, h. Poiret” and on the same label “1807 Meunier”. The attribution on the right-hand specimen was to the herbarium of Jean Louis Marie Poiret (1755–1834), a correspondent of Lamarck and co-author with him from 1797 on several volumes of “Encyclopédie Méthodique: Botanique” and other works.

### ﻿*Anemone stellata* – Lam.

The protologue of *Anemone stellata* Lam. (Lamarck 1783: 166) reads:

12. Anemone en étoile, *Anemone stellata*. Anemone foliis radicalibus tripartitis, lobis variè incisis, subtus venosis, petalis linearibus stellatim dispositis [Anemone with basal leaves divided into three parts, with lobes more or less incised, veined below, petals linear, arranged in a star-like pattern]. Anemone geranii rotundo folio, purpurascens [Anemone with rounded leaves like a *Geranium* L., slightly purplish] Bauh. Pin. 173. Tournef. 276. *Anemone hortensis latifolia* 3. Clus. Hist. 1. p. 249. *Anemone* 1. Dod. Pempt. 434. *Anemone* Hall. Helv. No. 1152. *Anemone hortensis* Lin. “Excluso Casp. Bauhini synonymo primo”.

Translation from French: “Although this species is very pretty, it is rarely cultivated in gardens; the preceding one [*A. pavonina*] is seen more often. This one has petals that are not as narrow and makes up a better-appearing flower, with more brilliance. Its root is tuberous, knotty, furnished with fibres, and has a slender, light and single-flowered stem. The basal leaves are borne on very long petioles, composed of three wedge-shaped leaflets, incised more or less deeply, veined and deflated, and provided at the end of their lobes with a small specific point. Some of these leaves are narrowly incised. The flower is terminal, either flesh-coloured, or red, or purplish, and composed of nine to fifteen narrow, linear petals, five to eight lines long [= 1.1–1.8 cm], coloured internally, whitish and a little hairy on their back, and which form a star shape by their arrangement. The collar is made up of three small narrow leaves, one of which is slightly cut. This plant grows in the stony and sterile places of Languedoc, Provence, Switzerland and Italy. It was communicated to me by Mons. Abbé Pourret. (v. s.). It flowers in March” (Lamarck 1783: 166).

Lamarck stated that he had received dried material of this taxon (v. s. = vidi siccam) from Pierre-André Pourret (1754–1818), who was born in Narbonne. After ecclesiastical studies, Pourret studied at the Jardin du Roi in Paris, which was then under the care of its intendant Georges-Louis Leclerc, Comte de Buffon (1707–1788). Thereafter, Pourret had maintained correspondence with the great naturalist scholars of his time, including Linnaeus, Jean-François Séguier in Nîmes, Philippe-Isidore Picot de Lapeyrouse in Toulouse and Carl Ludwig Willdenow in Berlin.

For the years 1787 and 1788, Pourret directed the natural history cabinet in Paris belonging to two brothers of the *ancien régime*, who both died in the Reign of Terror during the French Revolution (Bonnet 1916). The elder brother, Étienne Charles de Loménie de Brienne (1727–1794), was the Bishop of Condom and later Archbishop of Toulouse. His brother was Lieutenant-General Louis-Marie-Athanase de Loménie de Brienne (1730–1794).

In 1789, at the start of the French Revolution, Abbé Pourret left Narbonne and emigrated to Barcelona, where he was appointed director of the botanical garden and professor of natural history at the University of Barcelona. He became deputy director of the Botanical Garden of Madrid and then obtained a canonry at the Cathedral Church of Ourense in Galicia and canon-treasurer of the Metropolitan Church of Santiago de Compostela, where he gave lessons in botany. When he died in Santiago in 1818, he bequeathed his herbarium to the school of pharmacy in Santiago, which was then acquired by Complutense University in Madrid (MAF). There are no specimens of *Anemone stellata* among his collections in MAF (Paloma Cantó pers. comm.). There is, however, a specimen in Paris that was acquired from the “Collection de l’Abbé Pourret, extraite de l’Herbier légué de M. le Dr. Barbier 1847”. The surgeon Joseph-Athanase Barbier (1767–1846) had acquired the personal herbarium of the Abbé Pourret, which was initially conserved by Pourret at the château de Brienne for the brothers Loménie de Brienne. This was then moved to the cabinet of natural history in Paris (Bonnet 1916: 281, 283). The sheet, which has three flowering specimens, each with narrowly lanceolate tepals, as well as another specimen in fruit [P03181989], has two annotated labels on it and “Anemone decapetala L.” written on the sheet. The first label states, “Anemone stellata Lam. M. de Lamarck qui nous cite pour tenir cette plante de nous nous pavoir l’avoir malconnuè” [Mr de Lamarck who quotes us as having (received) this plant from us, (but) we must have misunderstood him]. The second has in Pourret’s hand “Anemone coronaria [crossed out] apennina [inserted instead] ex Seguiero et ex Gouano sola hortensis varietas cui synonymiam Tournefortii refert anemone geranii folio rotundo Tour. 276”.

When describing *A. stellata* Lamarck cited Linnaeus’s *A. hortensis* in his synonymy and moreover, did not refer to any specific material. The name *Anemone stellata* Lam. is therefore, not only illegitimate but it is also automatically typified by the type of *A. hortensis* (Art. 7.5). Haller’s description of Hal. 1152 which was also cited by Lamarck “Anemone seminibus lanatis, foliis radicalibus trilobis & multifidis, caulinis ovato lanceolatis” (Haller 1768) was described by Haller as having “petala novem” and “Flore miniato” and “purpureo flore, extus hirsuto”. It is clear he was describing variation within *A. hortensis*. There is a specimen collected by Haller conserved in G with the locality cited by Haller (1768) written on the label “Supra Moutru in dumetis” Hall. n. 1152. Anemone hortensis L.” [G00144586].

Lamarck had described his concept of *A. hortensis* in his earlier work (Lamarck 1779: 321) with the statement “La fleur est terminale, grande, lègérement purpurine, & composée de neuf pétales longs, étroits, marqués de quelques lignes & un peu velus en-dessous.” [The terminal flower is large, lightly purplish and composed of nine long, narrow petals marked with a few lines and the underside is a little velvety]. He went on to say that it grew in barren places of Provence and that some beautiful varieties are cultivated in gardens which have petals that are not as narrow [as the wild plant] and with much more vibrant colours (Lamarck 1779: 322).

#### ﻿Lamarck’s herbarium material

There are two sheets of *Anemone stellata* in Lamarck’s Herbarium in Paris. The first is of a single flowered specimen with ten intact narrow lanceolate tepals each with three straight linear veins above an involucre of three lanceolate leaflets, one split into two parts [P00282071]. It is likely to have been given to Lamarck by Pourret from a collection by his fellow Catholic monk Dom. Philippe François Emmanuel Fourmault (1728-c.1790?) who was a Benedictine monk based in Saint-Jean-d’Angély, Charente-Maritime in central-western France. Originally from Arras in the Pas-de-Calais region of northeast France, Fourmault botanised extensively across many parts of France notably in Burgundy. He was a correspondent of Lamarck and Jean-François Séguier in Nîmes. There is also a letter from Pourret to Séguier dated 4 November 1776 in Séguier’s correspondence which indicates that Pourret and Fourmault were in communication with each other (https://nakala.fr/10.34847/nkl.27a4u083).

The sheet has a label written in Lamarck’s hand “Anemone apennina d. Fourmault” and another label with “Anemone hortensis, latifolia simplici flore, III. clus. hist. p. 249.” and on the same label beneath that “hb Lamarck” and “Anemone stellata, encycl.”. The second sheet comprising another plant with 12 narrowly lanceolate tepals, is simply labelled “*Anemone hortensis* L.” and the Herb. Mus. Paris label stating Herbier de Lamarck acquis en Novembre 1886 [P00282072]. There is a faint bluish colouration where the tepals join the pedicel. The Pourret specimen [P03181989] comprising three flowering plants one with nine, the other two with twelve narrow tepals and a fourth plant in seed perfectly represent *A. hortensis*. Another specimen from the Herbarium of Jacques Cambessèdes (1799–1863) in Montpellier, has a label with “anemone Pourret 77” written on it with Anemone “apennina” crossed out and “stellata Lam.” added [MPU521269].

### ﻿Anemone pavonina var. fulgens DC.

In his protologue for the name Anemone pavonina var. fulgens Augustin Pyramus de Candolle (1778–1841) mentions “Gay ined.” (Candolle 1824: 18). This is a reference to Jacques Étienne Gay (1786–1864) a Swiss botanist from Prangins near Nyon c. 30 km from Geneva who worked extensively in Paris until 1848. Gay is known to have communicated with Candolle after the latter’s return there in 1816 and paid visits to Candolle in Geneva when visiting his family in Prangins (Candolle 1862: 411). There is, however, no extant correspondence specifically with respect to *Anemone* between Gay and Candolle in the archives in G, nor any extant specimens in G from Gay referring to this name (G. Barreira and P. Bungener pers. comm.). Candolle was uncertain of the status of this variety, describing it with a question mark and the statement “flores ampliores quam var *a* (v. s.)” [flowers larger than in var. *a*], the var. *a* being a reference to Lamarck’s *A. pavonina* i.e. a direct reference to plants with double flowers and the “v. s.” referring to the fact that he had only seen herbarium material (Candolle 1824: 18).

Candolle had already referred to the existence of both the single and double flowering states of this plant in his description of the double-flowered *A. pavonina* in an earlier work (Candolle 1818). In that he stated (translated from Latin): “It [i.e. *A. pavonina*] is rarely seen with a single flower; the variety with double-flowers is quite common in gardens under the names A. oeil de paon, Candiote, A. de Crète. Linnaeus referred to this in his synonymy, but in his herbarium, I found it among the varieties of *A. coronaria*; it is easily distinguished by the flower being surrounded by very acute sepals” (Candolle 1818: 198). Candolle included references to three collections “Hab. in vineis Vasconiae prope aquas-Tarbellicas (Dax) *Thore*; In Gallo-provincia circa Olbias (Ziz); Nicaeam *Risso* et verisimiliter in Oriente. ♃ fl. vere (v.s.sp. [vidi siccam spontaneum = I have seen it in a dried state from the wild] et v.c. [vidi cultam = I have seen it in cultivation])” (Candolle 1818).

The first collection mentioned by Candolle is to Jean Thore (1762–1823) a physician and botanist from Dax in the Landes. The locality cited by Candolle for Thore’s collection being vineyards near the Bronze Age hot spring settlement of the Tarbelli people, the foundation of the town of Dax. The second reference is to a collection by the German botanist Johann Baptist Ziz (1779–1829) from the archaeological site of Olbia, now known as Hyères, which sits on the coast between Marseille and Cannes in Provence. The third collection was by Joseph Antoine Risso (1777–1845) from near Nice.

Candolle in his later work described the species *Anemone pavonina* as having “sepalis 10 – 12 lanceolatis acutissimis” however, he also cited Morison’s illustration of a plant with multiple tepals (Morison 1680) and explicitly excluded two references “A. hortensis Thor. chl. land.: 238” and “A. pavonina Lois. not. 87” (Candolle 1824). Candolle then described his new var. fulgens with a question mark: “*b*var. fulgensfoliis tripartitis, lobis cuneatis inciso-dentatis, involucralibus sessilibus oblongis integris subincisisve, sepalis oblanceolatis apice latioribus basi attentuatis [*b*var. fulgens with tripartite leaves, bases cuneate, lobes incised-dentate, involucral leaves sessile, oblong, entire or subincised, sepals oblanceolate, broader at the apex and attenuated at the base]. Candolle specifically included in var. fulgens the two references that he had excluded from *A. pavonina* above: “*A. hortensis* Thor. chl. land.: 238” and “A. pavonina Lois. not. 87” (Candolle 1824). He added v. v. et s. = vidi vivam et siccam “I have seen living and dried plants”.

The first reference is to Thore’s “Essai Chloris Landes” (Thore 1803) in which Thore states “Corol. d’un rouge très vif: poly-pétale.” The second is to Jean-Louis-Auguste Loiseleur-Deslongchamps (1774–1849) who included his concept of *Anemone pavonina* in “Notice sur les plantes à ajouter à la Flore de France” in which he describes the flowers as differing from *Anemone hortensis* by their larger size and that the corollas have 10 to 15 petals. He stated “Elle croît dans les vignes à St. Pandelon près de Dax, où elle a été trouvée par M. Thore qui me l’a communiquée; j’en ai aussi reçu des échantillons de M. Grateloup” (Loiseleur-Deslongchamps 1810). Loiseleur-Deslongchamps also mentioned that there are plants cultivated in gardens with double flowers (Loiseleur-Deslongchamps 1810). Both the descriptions by Thore and Loiseleur-Deslongchamps refer to plants with large, red flowers with 10 to 15 tepals and not to plants with multiple numbers of tepals.

#### ﻿Candolle’s original material

There is a sheet in Loiseleur-Deslongchamps Herbarium [AV0022164] in the Muséum Requien-Avignon with two different collections on it both with flowers with single tepals. The right-hand specimen (Herb. Lois. 000.543) is a plant with thirteen red tepals. There is a label annotated “Anemone hortensis. Les coteaux de St Pandelon pres Dax, Printemps M. Thore 1808”. There is also on the same sheet another plant on the left (Herb. Lois. 000.542) with a single flower with twelve red tepals on it and a label annotated “Anemone pavonina Lois. in arvis St. Pandelon circa urbam aquaeus. M. Grateloup 1810”. The latter refers to a collection by Jean-Pierre Sylvestre de Grateloup (1782–1861) a physician in the town of Dax. Grateloup wrote a paper on the variations he had observed in what he called *Anemone pavonina* (Grateloup 1820). Both constitute original material cited by Candolle (1824). The left-hand specimen is selected here as the lectotype for the name Anemone pavonina var. fulgens DC. Another specimen in Loiseleur-Deslongchamps Herbarium (Herb. Lois. 000.544) [AV0022165] comprises two plants with single-flowers partly destroyed and another plant with multiple tepals but both are lacking any metadata.

There is a sheet in Gay’s own Herbarium now in K, which was acquired by Sir Joseph Hooker in 1868. The sheet has two pressed specimens alongside each other, one with flowers with multiple tepals and the other is a specimen with a large flower having eleven red tepals [K003266487]. The single-flowered specimen has a label with the annotation “in vinetis prope Dax, Grateloup misit Octobri 1817”. Attached to the sheet is a long descriptive note in Gay’s hand. Gay stated at the end of the description “Dans les vignes des environs de Dax. Envoyée par Mr. Grateloup, en Octobre 1817 sous le nom d’Anemone pavonina Lam.” [K003266487]. There is no evidence, however, that Candolle had seen these specimens.

There are three sheets in Candolle’s own Herbarium although none were specifically cited in the protologue. These were collected by Jean-Marie Léon Dufour (1780–1865) who was also a French medical doctor, botanist and entomologist from Saint-Sever in the Landes, c. 45 km east of Dax. Léon Dufour first encountered Candolle while they were both medical students in Paris in 1804. They met on an excursion with other naturalists in the forest of Fontainebleu and maintained correspondence after Dufour’s return to Saint-Sever as a practicing physician in 1806. In September 1807 Candolle visited Léon Dufour on his way to explore the Pyrenees. Candolle had been appointed professor of botany in the medical faculty of Montpellier University in 1807 and in January 1808 was appointed director of the Jardin des Plantes de Montpellier. He remained in Montpellier until 1816 when he returned to his native Geneva (Hans Walter Lack pers. comm.). Dufour’s main herbarium is conserved in the Herbarium du Jardin botanique de Bordeaux (BORD). The sheet G00144566 has two single-flowered specimens on it, one with ten and the other with twelve tepals. The sheet is annotated “A. fulgens Gay ined. Anemone pavonina simple et double (voy. A. pavonina) a.’s vignes dela chalosse Mr Dufour 1818” written in de Candolle’s hand. HERB.PRODR. (G-DC) [G00144566]. SIB no. 149786/1. The sheet G00144569 has three different collections on it by different collectors, all comprise double flowering plants. The left-hand specimen is annotated “Cet echantillon provient des vignes de la Chalosse ou il est sauvage. M’s. L. Dufour qui me l’a donné en 1818 assure que c’est l’individu a fleurs doubles de l’ A. stellata var. fulgens mais je ne puis l’admetra avec certitude a n’ayant point d’intermediaires” [“This sample comes from the vineyards of Chalosse where it is wild. Mons. L. Dufour, who gave it to me in 1818, asserts that it is the individual with double flowers of A. stellata var. fulgens but I cannot admit it with certainty since I have no intermediaries] written in de Candolle’s hand. “Dufour 1818” is written in another’s hand (M. Callmander pers. comm.). HERB.PRODR. (G-DC) [G00144569]. SIB no. 149782/1. The sheet G00144542 has three single flowered specimens on it two of which were both collected by Léon Dufour (G00144542 SIB no. 149812/1). The left-hand specimen with thirteen tepals has written on the label “Vignes du Montmarsan Mr. Dufour 1818” [G00144542]. The right-hand specimen with twelve tepals has “Anemone stellata coul. non chang. la ne (illeg.??) vignes de Montmarsan Mr Dufour 1818” [G00144510].

These three sheets confirm Candolle’s uncertainty as to the status of his Anemone pavonina var. fulgens DC. The first sheet [G00144566] comprises two flowering specimens with simple flowers both with 12 tepals. Candolle states on this sheet “voyez [see] *Anemone pavonina*” immediately after “double” which indicates that he did not equate the single-flowered variety which he called var. *a* on this sheet to represent *Anemone pavonina* (i.e. var. pavonina). The second sheet comprises a mixed collection of three different gatherings of plants with multiple tepals collected by different individuals. The left-hand specimen [G00144569] was gathered by Léon Dufour from the same locality near Dax. Candolle labelled that plant with multiple tepals Anemone stellata var. fulgens. The third sheet only has plants with twelve and thirteen tepals.

There is also a sheet in Paris with a single collection on it comprising a flower with multiple tepals. There is a label which simply states “Anemone pavonina. B. G. 27. Nica” and another label has “Herbier de la Flore Française (Bot. Gall.) donné au Muséum par A. P. de Candolle. 1822” [P03182116]. In conclusion Candolle believed that large-flowered plants with a few tepals referred to his var. fulgens while plants with many narrow tepals referred to var. pavonina.

### ﻿Anemone hortensis var. stellata Gren. and Godron.

Jean Charles Marie Grenier (1808–1875) and Dominique Alexandre Godron (1807–1880) together compiled and wrote the comprehensive “Flore de France” in three volumes from 1847 to 1856. In the first volume they included *Anemone hortensis* including three additional varieties (Grenier and Godron 1847). They defined *A. hortensis* with the description: “Calice à 10–12 sépales et plus, *glabres extèrieurement*, obovales ou lancéolés, plus ou moins aigus, ou enfin sublinéaires très-aigus” [Calyx with 10 – 12 sepals or more, externally glabrous, obovate or lanceolate, more or less acute or sublinear and very acute]. Their first variety var. *a stellata* they described as: “Sépales 8–10, lancéolés, obtus parfois apiculés [Sepals 8–10, lanceolate, obtuse or sometimes apiculate]. *A. hortensis* (de presque tous les auteurs). *A. stellata Lam. Enc. 1. p.* 166.” They stated the distribution for var. *a* was: “Grasse, Fréjus; Navarreins (Basses-Pyrénées); Dax”. Their var. *β fulgens* they described as: “Sépales 8–10, grands, obovales, en coin à la base, élargis au sommet obtus, parfois apiculés [Sepals 8–10, large, obovate, narrowed at the base, widened at the obtuse apex, sometimes apiculate]. *A. hortensis Thore chl. land*. 238; *A pavonina Lois. gall.* 1, *p.* 400; *Rchb. l. c.* f. 4650”. Distribution stated as: “across southern France and Corsica”. Their third var. *γ pavonina* was: “Sépales très-nombreux, lancéolés-linéaires, très aigus [Sepals very numerous, linear-lanceolate, apex very acute]. *A. pavonina D C. fl. fr* 5, *p.* 634; *Dub. bot.* 1 *p.* 5”. Distribution: “Dax, Saint-Sever; Grasse etc”.

Grenier and Godron when they published *A. hortensis* “*α stellata*”, were clearly distinguishing this variety from “the species”, i.e. from A. hortensis L. var. hortensis, indicated by their recognition of it as having a different number of sepals (Grenier and Godron 1847). Linnaeus in fact only alluded to numerous “petals” in most of his publications, mentioning “sepals nine” only once (Linnaeus 1771). Linnaeus’s type specimen however, consists of two flowering plants: one with ten tepals and the other with twelve. Grenier and Godron’s further statement “*A. hortensis* (de presque tous les auteurs [of almost all authors])” also indicated that they considered their var. stellata to be different from typical *A. hortensis* L. and their citation of “*A. stellata Lam. Enc.* 1, *p.* 166” can then be taken as explicitly excluding Lamarck’s synonym “*Anemone hortensis*. Lin.”, and thus the type of *A. stellata*. The name *Anemone stellata* Lam. being superfluous and illegitimate, is automatically typified by the type of *A. hortensis* (ICN Art. 7.5). Grenier and Godron’s A. hortensis var. stellata is based on a different type collected near Grasse as stated in the protologue. Original material was collected by Godron’s colleague Charles Joseph August de Baudot of Sarrebourg, and is designated as lectotype in this paper (Fig. 2A).

Grenier and Godron’s Anemone hortensis var. fulgens (DC.) Gren. and Godr. is a combination based on Candolle’s Anemone pavonina var. fulgens DC. (Candolle 1824). Although Candolle’s varietal name was not directly cited by Grenier and Godron (1847), it is nevertheless based on the same type according to their shared list of synonyms (see discussion and typification below).

A. hortensis var. pavonina (Lam.) Gren. and Godr. is also a new combination, although again it is only Candolle, and his contributing author Jean Etienne Duby, that were cited and not Lamarck. Both Candolle and Duby, however, included *A. pavonina* Lam. as a synonym in their own descriptions of *A. pavonina*, thereby permitting Grenier and Godron to validate the new combination (Candolle 1818, 1824; Duby 1828).

### ﻿Anemone stellata var. heldreichii Boiss.

Theodor von Heldreich (1822–1902), director of the Natural History Museum and of Queen Amalia’s Royal Garden in Athens (now the National Garden) collected a small, white or very pale pink-flowered *Anemone* on the island of Crete in 1846. This was described three years later as a variety of *Anemone stellata* Lam. by the Swiss botanist Pierre Edmond Boissier (1810–1885) as A. stellata var. heldreichii Boiss. (Boissier 1849) It had to wait another 51 years until the Vienna based physician of Hungarian descent Eugen von Halácsy (1842–1913), also called Jenö Halácsy, who worked closely with Heldreich, correctly identified it as a variant of *A. hortensis* (Halácsy 1900). It was later recognised as a species in its own right *Anemone heldreichiana* by the French mycologist Michel Gandoger in 1916 until the Austrian botanist Karl Heinz Rechinger (1906–1998) demoted it to the rank of subspecies within *A. hortensis* in 1943, stating (translated from German) “As a geographical race it deserves the rank of a subspecies”.

It is a plant restricted to montane slopes of Crete and a few adjacent islands, exhibiting small ternate leaves, each of the three lobes much divided and incised and with pale midveins on the adaxial surface. The flowers are white occasionally pinkish, with narrow, elliptic tepals streaked with three blue veins. The anthers are also notably blue (Fig. 2D). This taxon, although known under the plethora of taxonomic ranks outlined here, has not been sampled adequately in molecular analyses to ascertain its correct taxonomic status.

## ﻿Discussion

The establishment of a stable nomenclature and the correct taxonomic identity of the names referred to within this paper is of long-term importance to botanists, ecologists and horticulturists. The results from a large range of molecular and cytological data support the recognition of *Anemone hortensis* as a single species that includes material previously known as *A. pavonina*, *A. fulgens* and a plethora of other names (see synonymy below). *Anemone hortensis* can be segregated at the infraspecific level with the recognition of three varieties and one forma (see Taxonomic treatment below).

Linnaeus’s *A. hortensis* was rather poorly defined. His various descriptions and many references told of a species with nine or more tepals. The lectotype and syntype in the Clifford Herbarium are based on red-flowered specimens cultivated in the Netherlands with ten, eleven and twelve broadly obovate tepals (Fig. 1C). The material in the Linnaean Herbarium, however, has thirteen to sixteen more narrowly lanceolate tepals and more dissected leaves from wild material collected in Italy. The origin of the Stockholm specimen, also with lanceolate tepals, is unknown. Other than variation in leaf dissection and in the number and width of sepals, it is clear that Linnaeus recognised these as belonging to the same species.

It is very clear from Lamarck’s own description, his herbarium specimen and the large number of references and illustrations that he cited that he was basing his *A. pavonina* on plants with multi-tepalled or doubled flowers. Lamarck himself leaves no doubt about this, as he stated in his protologue, “quoique je ne l’aie vue qu’a fleurs doubles”, i.e. that at the time of compiling his description, he only knew plants with doubled flowers. His inclusion of Casper Bauhin’s entries I, IV, V and VI as synonyms (C. Bauhin 1623) under Bauhin’s heading of “Anemone flore pleno” [*Anemone* with double flowers] provides unequivocal confirmation of Lamarck’s understanding of the plants in question (Lamarck 1783). In addition, Lamarck made the assumption that, as *A. coronaria* was from the Levant, it was likely that the wild *A. pavonina* “la plante naturelle qui fait le type de cette espèce” [the wild plant which constitutes the typical (i.e. non-double) form of the species] was also from that region (Lamarck 1783). He undoubtedly read Clusius’s text for “*Anemone hortensis latifolio pleno flore 1*”, in which Anna-Maria de Heusenstain is credited with supplying the plant to Clusius in Vienna (Clusius 1601). Lamarck stated that this plant was in cultivation in the Jardin du Roi in Paris and, at the end of his protologue, added (v. v.), i.e. *vidi vivam*, meaning that he had seen the living plant.

It has only relatively recently been possible to examine Lamarck’s Herbarium in Paris, where the type is now conserved. Lamarck’s Herbarium was acquired prior to his death in 1829 by the physician Johannes August Christian Roeper (1801–1885), professor of medicine from 1827 at the University of Basel and from 1836 professor of natural history at the University of Rostock. Lamarck’s Herbarium consequently moved with Roeper and was acquired by the University of Rostock in 1875, eventually being sold on to the Muséum in Paris in 1886. During those movements, labels had become unattached, and some specimens were remounted. The double-flowered specimen (Fig. 1D) from Lamarck’s Herbarium [P00282070], albeit lacking any additional information on the sheet and having in all probability been gathered from a plant cultivated in the Jardin du Roi in Paris, is eligible as the lectotype (Art. 9.4) for the name *Anemone pavonina*. The central flowering stem with multiple tepals is designated as such here. Lamarck does not state the wild origin of the cultivated material, but it is possibly from the same source as the single-flowered specimen [P00282069], later indicated by an unknown hand to have been gathered from near Nice, where the red-flowered plants had long become naturalised. Both single- and double-flowered plants occur together extensively in that area from introductions that originated in the eastern Mediterranean. The date of introduction into Western Europe of those plants, and by whom, is also not known but may have been as early as the Late Middle Ages (c. 1300–c. 1500) to Liguria during the Genoese Republic. In summary then, Lamarck’s *A. pavonina* was undoubtedly a multiple-tepalled or double-flowered variant of *Anemone hortensis*.

Although Lamarck’s *A. stellata* is a synonym of *A. hortensis*, and his name is consequently based on the type of *A. hortensis*, it is significant in our context that he stated on the label of the specimen [P00282071] the reference to Clusius’s name “Anemone hortensis latifolia simplici flore”. This statement serves to confirm his comparison between the double-flowered plant that he had just described as *A. pavonina*, along with all its concomitant references, and the single-flowered *A. stellata*, which he recognised only in comparison to the double-flowered plant (see below for recognition of A. hortensis var. stellata).

Looking at all the material available to Candolle when determining the identity of A. pavonina var. fulgens , it appears that he was not basing his var. fulgens on plants with multiple tepals (which he eventually recognised as *Anemone pavonina* Lam.), but on those with broader tepals and a more vivid red colouring, which he had seen growing in gardens under the name *A. hortensis* – “Dont les pétales sont moins étroits et les couleurs beaucoup plus vives” [whose petals are less narrow and the colours much more vivid] (Candolle 1805). Candolle more or less repeated this statement later but had altered the name of the species from *A. hortensis* to *A. pavonina*, thereby instigating a confusion that has been persistently perpetuated. He stated: “L’anémone oeil de paon a la fleur beaucoup plus grand et d’une rouge très-éclatante. Elle a été trouvée par M. Thore dans les vignes de S. Pandelon près Dax: on en cultive diverses variétés doubles dans les jardins.” [the peacock’s eye anemone has a much larger flower (than *Anemone stellata* above), which is a very bright red. It had been found by Mr Thore in the vineyards of St. Pandelon near Dax: various double varieties are cultivated in gardens] (Candolle 1815). In his protologue he described the difference between his renewed concept of A. pavonina and his var. fulgens simply in the statement that his var. b has larger flowers than those in var. a: “Flores ampliores quam var. a” (Candolle 1824). This name, which clearly did not refer to Lamarck’s double-flowered plant, is consequently placed into the synonymy of *Anemone hortensis* L.

When it comes to the purported recognition of the putative hybrid *Anemone × fulgens*, it is highly probable that “within-species” hybridisation may have occurred when the eastern Mediterranean red-flowered A. hortensis var. hortensis was introduced into France and northern Italy and crossed with the extant lilac or purple-flowered A. hortensis var. stellata. A plethora of flower-coloured variants recognised by Jordan and others, at a range of botanical ranks, occur in southeast France. These are not, however, based on Candolle’s var. fulgens, as already explained above.

Gina Luzzatto mentioned that the plantsman Edward Augustus Bowles had presented drawings he had made in the south of France of what he believed were hybrids between *Anemone pavonina* and *A. stellata* at a meeting of the Royal Horticultural Society’s Scientific Committee on 27 March 1928 (Luzzatto 1933). These, and Bowles’s cultivated herbarium material, are now conserved in WSY, and many represent double-flowered plants. Luzzatto stated that Bowles posited that *Anemone fulgens* was probably of hybrid origin due to the presence of low levels of seed germination. Luzzatto went on to show that both single- and double-flowered forms were in fact found across southern France (Luzzatto 1933). Bowles later published his own historical account of these taxa, implying that *A. fulgens* was of hybrid origin, citing *A. hortensis* and *A. pavonina* as parents (Bowles 1948).

As has been explained above, *A. pavonina* is a double-flowered expression or teratological form of *A. hortensis* which has been found naturalised across southern France and northern Italy and occurs less frequently in its native habitat in Greece, although it does also occur there. The double-flowered expression has occurred on many occasions, as shown by the many gatherings seen in various herbaria from across the range of the species. There is consequently no evidence of interspecific hybridity, although doubling of floral parts at the expense of stamens would inevitably result in either lower levels of fertility in semi-double-flowered plants or no fertility at all in fully double-flowered plants.

Tutin, in the treatment of *Anemone* in “Flora Europaea”, stated that *A. fulgens* “is probably *A. hortensis × pavonina*. It is more or less intermediate and does not breed true”, but did not refer to Bowles’s paper (Tutin 1964). Davis et al., in their treatment of *Anemone* for “Flora of Turkey”, did refer to Bowles and also considered that *A. fulgens* was a hybrid between *A. hortensis* and their concept of *A. pavonina*, which they considered replaced *A. hortensis* in Eastern Europe (Davis et al. 1984). As already explained, *Anemone hortensis* originated in Eastern Europe.

Maïa and Venard (1976), in their study on putative hybrids between *A. hortensis* and the related *A. coronaria*, were only able to obtain one seedling from a sowing of 300 seeds, and, moreover, that single plant was not able to reach maturity (Maïa and Venard 1976). They also indicated that pollen fertility from six plants of A. pavonina var. ocellata had 95% normal fertility, and from seven plants of *A. hortensis* had between 80% and 95% normal fertility. Pollen fertility from the single plant of what they considered to be *A. pavonina × A. hortensis* was 99% normal, whereas pollen from *A. pavonina × A. coronaria* was 0% fertile. This indicated that the potential for hybridisation between these species was not achievable (Maïa and Venard 1976). Their conclusion that A. pavonina var. ocellata and *A. hortensis* are cenospecies, i.e. infraspecific taxa within *A. hortensis*, is in agreement with the molecular and morphological assessments of these taxa mentioned above (Maïa and Venard 1976).

Grenier and Godron’s Anemone hortensis var. stellata Gren. and Godr. was not based on Lamarck’s species name (a synonym of *A. hortensis*) and was validly published as the name of a new variety with narrowly lanceolate tepals (Fig. 2A, B). As such, it is accepted here, and a lectotype is chosen for the name. Anemone hortensis var. fulgens (DC.) Gren. and Godr., however, although a validly published combination, is considered to be a synonym of *A. hortensis*. The name Anemone hortensis var. pavonina (Lam.) Gren. and Godr., on the other hand, is the earliest validly published combination of that taxon at the rank of variety and is accepted here based on Lamarck’s type in P.

Boissier’s white-flowered Cretan Anemone stellata var. heldreichii, (Boissier 1849) collected by Heldreich, placed as a variety in *A. hortensis* by Halácsy, recognised as a species in its own right by Gandoger (as *A. heldreichiana*), and finally as a subspecies of *A. hortensis* by Karl Heinz Rechinger, needs further work to ascertain its status in relation to *A. hortensis*. It has not been sampled for molecular analysis, and consequently, for the time being, it is recognised, based on its morphology, as a variety of *A. hortensis*.

### ﻿Taxonomic treatment

#### ﻿Description of *A. hortensis* and key to the varieties

***Plants*** perennial, herbaceous, with non-stoloniferous, irregular, branching, rhizomatous tubers 2–6 × 1–2 cm; ***basal leaves*** dimorphic, petioles 5–10 cm long with broad stipule-like bases, primary leaves palmately-trisected 2–4 × 3–6 cm., margins dentate, slightly pubescent to glabrous above, pubescent beneath, secondary leaves palmate, finely dissected into 3–5 (–7) toothed lobes; ***stems*** erect, 10–30 (– 40 cm.), single-flowered, covered in short silvery hairs, bearing three, lanceolate, acute, sessile, entire leaflets 1–2 × 0.4–0.8 cm., some distance below the flower forming a collarette or involucre, each leaflet occasionally further divided or serrated, with 1–3 veins; ***flowers*** solitary, 3–6 (– 8) cm. in diameter, radially symmetrical, with (8 –) 12–18 (– 50+) bluish-mauve, lilac, pink, purple, red or white, petaloid, free, sometimes overlapping tepals, each with (1–) 3–5 primary red or blue veins, each tepal (0.3 –) 1.5–4.0 × (0.5–) 1.2–2.0 cm., elliptic, linear, lanceolate or obovate with either acute, acuminate or obtuse apices; ***stamens*** numerous, filaments dark blue or violet, anthers blue; ***ovary*** superior; ***fruits*** ovoid, lanate, carpels 0.5–1 mm long, styles straight, achenes congested into a compact infructescence, each achene ovoid, 2.9–3.2 × 1–1.7 mm with villose hairs 3.5–5 mm long; 2n = 16; flowering March to May.

Owing to the enormous amount of variation exhibited by this species across its distributional range, this key is as accurate as it can be, with the caveat that many exceptions in e.g. numbers of floral parts and floral colours also exist, as do intermediate states. Having said that, it seems sensible to recognise four well-defined varieties. The red-flowered var. hortensis is restricted to the eastern Mediterranean (naturalised in southern France and northern Italy), while the lilac or purple-flowered var. stellata occurs throughout the range of the species but is less common in the east. Plants with multiple numbers of narrow tepals are recognised as var. *pavoninа*, and the white-flowered Cretan and local island endemics as var. heldreichii.

**Table:** 

1a	Flowers red, rarely purple, tepals 8–12, obovate, 1.5–3.2 × 1.2–1.8 cm with 3–5 veins, apex acute or obtuse	**Anemone hortensis var. hortensis**
–	Tepals 12–20 (– 50+)	**2**
2a	Flowers with tepals 12–20, lanceolate or elliptic	**3**
–	Flowers with tepals 20–50+ narrowly linear 1.5–3.2 × 0.3–0.8 cm with 1 (– 3) veins, outer tepals rarely calycoid, green	**Anemone hortensis var. pavonina (Lam.) Gren. and Godr.**
3a	Flowers lilac, purple or rarely red; tepals narrowly elliptic or linear-lanceolate, 1.8–4.0 × 0.5–1.2 cm with 3 veins, apex acuminate	**Anemone hortensis var. stellata Gren. and Godr.**
–	Flowers greyish-white or pale pink, tepals elliptic-lanceolate 1.2–1.8 (– 2.0) x 0.8–1 cm, with 3 veins, apex obtuse to acute	**Anemone hortensis var. heldreichii (Boiss.) Halácsy**

### ﻿Nomenclatural treatment and synonymic conspectus

#### ﻿ Anemone hortensis

Taxon classificationPlantaeRanunculalesRanunculaceae

L. Sp. pl. 1: 540 (1753)

4404F7F5-6AB0-53B7-A3ED-FC1666AEF98A

Fig. 1C

Anemone hortensis L. Sp. pl. 1: 540 (1753) Lectotype designated by Arne Strid in Jarvis et al. (ed.) Taxon 54: 469 (Jarvis et al. 2005): Herb Clifford: 224, “Anemone hortensis, Pulsatilla 4, sheet A” (lecto. BM!) [BM000628826].
≡ Anemone stellata Lam. Encycl. 1(1): 166 (1783) nom. illegit, typified by the type of A. hortensis L.
≡ Anemone versicolour Salisb., Prodr. Chapel Allerton: 371 (1796).
≡ Anemone stellata var. versicolour (Salisb.) Sweet, Br. fl. gard. 2: sub t. 112 (1825).
≡ Anemone hortensis subsp. stellata (Lam.) Nyman, Consp. Fl. Europ: 3 (1878). (nom. illegit Art. 53.3). Typified by the type of A. stellata Lam., which is the type of A. hortensis L. (see note 1 below).
≡ Anemone hortensis [forme] stellata (Lam.) Rouy and Foucaud, Fl. France 1: 48 (1893) nom. inval. (see note 2 below).
(≡ Anemone hortensis [forme] stellata (Lam.) Albert, Cat. pl. vasc. Var: 4 (1908) nom. inval.).
= Anemone formosa E.D.Clarke, Travels Eur., Asia & Africa 2(1): 145 (1812). Type: Turkey, Troas [Biga], Mt. Gargarus [Gürgen Daği], 10 March 1801, *E.D.Clarke* s.n. (type n.v.).
= Anemone pavonina var. fulgens DC. Prodr. 1: 18 (1824) Lectotype designated here (Art. 9.3): France, Landes, “Anemone pavonina Lois. in arvis St. Pandelon circa urbam aquaeus. *J-P. S. de Grateloup s.n.* 1810” (Herb. Lois. 000.543) (lecto. AV!) [AV0022164]; syntype “Anemone pavonina, mars, avril, environs a Dax. M[isit] *Grateloup* s.n.” (Herb. Lois. 000.547 (L4)) (syn. AV!) [AV0022166].
≡ Anemone fulgens (DC.) Rchb. Iconogr. Bot. Pl. Crit. 3: 1. t .201, f. 343 (1825).
≡ Anemone hortensis var. fulgens (DC.) Gren. and Godr. Fl. France 1: 14 (1848).
≡ Anemone hortensis subsp. fulgens (DC.) Nyman, Consp. Fl. Eur.: 3 (1878) (see note 1 below).
≡ Anemone hortensis subvar. fulgens (DC.) Hayek, Repert. Spec. Nov. Regni Veg. Beih. 30: 319 (1924).
= Anemone stellata var. purpurea Sweet, Br. fl. gard. 2: t. 112 (1825) Lectotype designated here (Art. 9.12): Sweet, Br. fl. gard. 2: [Icon] t. 112.
= Anemone latifolia Bellardi ex Re, Mem. Reale Accad. Sci. Torino 33: 233 (1829) “Habitat in agro Nicaensi in locis incultis”. Lectotype designated here (Art. 9.12): [Icon] “Anemone latifolia ex coccineo phaenicei coloris, unguibus parvis subpallidus” (Chabrey 1666: 462; see note 3 below).
= Anemone hortensis var. obtusiflora Spach, Hist. Nat. Veg. 7: 251 (1839) Lectotype designated here (Art. 9.12): [Icon] Bot. Mag. 3–4: t. 123 (1790–1791).
= Anemone hortensis var. acutiflora Spach, Hist. Nat. Veg. 7: 251 (1839). Lectotype designated here (Art. 9.12): [Icon] Reichenbach, Iconogr. bot. crit 3: t. 201 f. 343 (1825).
= Anemone hortensis var. alba Risso, Flore de Nice: 7 (1844) Type n.v.
= Anemone hortensis var. variabilis Risso, Flore de Nice: 7 (1844) Type n.v.
= Anemone hortensis var. purpurascens Risso, Flore de Nice: 7 (1844) Type n.v.
= Anemone hortensis var. zonata Risso, Flore de Nice: 7 (1844) Type n.v.
= Anemone bauhinii Risso, Flore de Nice: 7 (1844) Lectotype designated here (Art. 9.3): France, Nice “Col de Villefranche prairies”. Collector unknown. Reçu au Muséum [Paris] en 1955 (lecto. P!) [P02467502].
= Anemone bauhinii var. alba Risso, Flore de Nice: 7 (1844) Type n.v.
= Anemone bauhinii var. ferruginea Risso, Flore de Nice: 7 (1844) Type n.v.
= Anemone lepida Jord., Bull. Soc. Linn. Lyon ser. 2 vol. 7: 427 (1861) Neotype designated here: France, Provence, “Sous les oliviers, Grasse” *C.Bertrand* s.n. 11 March 1906 (neo. P!) [P02695662].
≡ Anemone purpurata Jord., Bull. Soc. Linn. Lyon ser. 2 vol. 7: 427 (1861).
≡ Anemone hortensis var. lepida (Jord.) Ardoino, Fl. Anal. Alpes-mar.: 13 (1867).
≡ Anemone hortensis [forme] lepida (Jord.) Rouy & Foucaud, Fl. France 1: 49 (1893), (nom. inval. See note 2).
= Anemone variata Jord., Bull. Soc. Linn. Lyon ser. 2 vol. 7: 427 (1861) Lectotype designated here (Art. 9.12): France, “Anemone versicolour, Grasse et Mouhans [Mouans-Sartoux] (Var) 1844. *J-L. Hénon* s.n. ex Herb. Al. Jordan 187, Herbier Bonaparte (stamp)” (lecto. LY!) [LY0049050]; isolectotype: France, “Anemone versicolour J., Grasse et Mouans (Var) 1844, M. Hénon, ex Herb. Al. Jordan 187” (isolecto. MPU!) [MPU521442]; syntype: “Anemone versicolour Jord. Cannes, Alpes Maritimes, rec. Loret, 1853, *Jordan* s.n. s.d. ex Herb. Al. Jordan 187” (syn. P!) [P03181955]; syntype “Anemone versicolour var. fl. rubro-pallida a Grasse (Var) in horto mea legi janvier 1853. Alexis Jordan 1853” (syn. NCY!) [NCY0087143]; syntype “Anemone versicolour Jord. Du Var mihi 29 Mai 1853” (syn. LY x 2!) [LY0799593; LY0799594]; syntype “Anemone versicolour Jord. Cannes (Alpes Maritimes n Lorn 1853” (syn. LY!) [LY0799587].
≡ Anemone hortensis var. variata (Jord.) Ardoino, Fl. Anal. Alpes-mar.: 13 (1867)
≡ Anemone hortensis [forme] variata (Jord.) Rouy and Foucaud, Fl. France 1: 49 (1893), (nom. inval. note 2).
= Anemone fulgens var. purpureo-violacea Boiss. Fl. Orient. 1: 12 (1867) Lectotype designated here (Art. 9.3): Turkey, “Anemone formosa Const.” Constantinople [Istanbul], 1837, *Aucher-Eloy* 10 (lecto. P!) [P03180155]; isolectotype: Turkey “Byzantium” Aucher-Eloy Pl. Orientales, *Aucher-Eloy* n.10 (right-hand specimen isolecto. P!) [P03182168]; syntype: Greece, “Anemone stellata entre Nisi [Messini] et Colomata [Kalamata] sur les bains Romains, Morée [Peloponnese] Gittard s.d. s.n.” Herb. G-BOISS label. (syn. G!) [G00788455] SIB-457127/1; syntype: Greece, “Anemone hortensis b. Attica”, Spruner s.d. s.n. (Herb. Fl. Orientalis G-BOISS label) (syn. G!) [G00788454] SIB-457126/1; syntype: “Graecia. Mr W Spruner 1840” (syn. G!) [G00788446] SIB-457118/1.
≡ Anemone pavonina var. purpureo-violacea (Boiss.) Halácsy, Consp. Fl. Graec.1: 5 (1900).
≡ Anemone hortensis subvar. purpureo-violacea (Boiss.) Hayek, Repert. Spec. Nov. Regni Veg. Beih. 30: 319 (1924).
= Anemone stellata var. grandiflora Pons, Bull. Soc. Bot. Fr. 30: lxxxii (1883) Provence, “Le Bar, Mouans, Grasse, dans les cultures”. Lectotype designated here (Art. 9.3): France, “Le Bar quartier des vignes” l’Abbé *A.Pons* s.n. 20 January 1881 (lecto. MPU!) [MPU532042] (see note 4 below).
≡ Anemone hortensis subvar. grandiflora (Pons) Burnat, Fl. Alp. Marit. 1: 13 (1892).
≡ Anemone hortensis [forme] grandiflora (Pons) Rouy and Foucaud, Fl. France 1: 48 (1893), nom. inval. (see note 2 below).
≡ Anemone hortensis var. grandiflora (Pons) P.Graebn., Syn. Mitteleur. Fl. [Ascherson and Graebner] 5(3): 8 (1935).
= Anemone stellata var. parviflora Pons, Bull. Soc. Bot. Fr. 30: lxxxiii (1883) Lectotype designated here (Art. 9.3): France, Grasse, Le Bar, Mouans *A.Pons s.n.* Fevr-Avril 1880 (lecto. K!) [K003266312].
≡ Anemone hortensis subvar. parviflora (Pons) Burnat, Fl. Alp. Marit. 1: 13 (1892)
= Anemone pavonina var. occidentalis Luzzatto, Arch. Bot. Sist. 9(3–4): 208 (1933). Lectotype designated here (Art. 9.3): France, Basses Pyrenées, Gan, “vignes des coteaux argileux”, 20 April 1882, *E.Doassans* 4016 det. Dr Gina Luzzatto mihi, 24.3.1933 (lecto. P!) [P03182081]; (isolecto. BR!) [BR0000031559218]; (isolecto. P x 2!) [P00266532; P02840556]; syntype: (left specimen only): France, “St. Pandelon in Vasconia”, *E.Camus* 177a, s.d. det. Dr Gina Luzzatto mihi 24.3.1933 (syn. P!) [P03182079]; syntype: France, Basses Pyrenées, “dans les vignes près Pau” *F.Schultz* s.n. det. Dr Gina Luzzatto mihi 30.1.1933 (syn. B!).
= Anemone pavonina f. decolorata Luzzatto, Arch. Bot. Sist. 9(3–4): 208 (1933) Lectotype designated here (Art. 9.3): France, “Anemone stellata Lam. environs St Sever Dept. Dax, Landes comm. D. L. Dufour” (the year 1868 no. 23 added later). Det. Dott. Gina Luzzatto, 24 March 1933. (lecto. P!) [P00266535].
- Anemone stellata var. primigenia A.Gubler, Bull. Soc. Bot. France 8: 243 (1861) nom. nud.

##### Distribution and habitat.

Anemone hortensis var. hortensis is found in Albania, Bulgaria, Greece, North Macedonia, and Turkey in Europe and Anatolian Turkey, growing in open meadows or among shrubs, sometimes in woodland clearings from 200 to 1200 m. Ziman et al. (2011) noted the presence of seasonally dimorphic leaves in *A. hortensis*. In early spring they observed that trisected leaves occurred with only a few obtuse lobules, whereas in late spring more divided leaves emerged with numerous acute lobules. These authors also indicated that for *A. coronaria*, both basal and involucral leaves were much more finely divided and dissected throughout the growth cycle of the plant (Ziman et al. 2011). Flowering in March and April. The forma regina (Risso) J.Compton (see below) with golden yellow or white at the base of each red or purple tepal, which collectively form the “queen’s” crown in the centre of the flower, is most frequently found (Fig. 2C), whereas flowers which are entirely red or entirely purple are less common.

##### Note 1.

In the Foreword to “Conspectus florae europaeae”, Carl Fredrik Nyman stated, “Subspecies, litteris minimis impressae, asterisco notantur; varietates linea longiora ante nomen” [Subspecies printed in small letters are marked with an asterisk; varieties with a longer line before the name] (Nyman 1878: Foreword). The correct combination at the rank of subspecies is therefore Anemone hortensis subsp. fulgens (DC.) Nyman.

##### Note 2.

Heywood (Heywood 1958: 89–93) succinctly considered the status and application of the names of the infraspecific taxa included in Rouy and Foucaud’s “Flore de France” (Rouy and Foucaud 1893–1913). In our context this discussion is pertinent to the names provided by Rouy and Foucaud in vol. 1; highly relevant is their use of the term “forme”, which in their terminology denoted a rank intermediate between variety and subspecies i.e. above that of variety (Rouy and Foucaud 1893: xi–xiii). They make it quite clear as to how they propose to use the term in their work: “Ajoutons qu’il nous a paru nécessaire d’indiquer par des caractères typographiques différents la valeur plus ou moins grande des plantes au point de vue du groupe spécifique: espèces, sous-espèces, formes, variétés, sous-variétés.

Une innovation qui sera probablement remarquée, c’est la valeur que nous attribuons à la forme, que nous considérons ici comme synomyme de la race en horticulture…….Nous estimons donc la forme d’un degré supérieur dans l’échelle de la classification à la variété” (Rouy and Foucaud 1893: xi). Art. 4.1 of the ICN states clearly that forma sits below the rank of varietas. Their usage of the term “forme” consequently contradicts both Art. 5.1 and Art. 37.6 of the ICN, and their unorthodox use of that term either for new names or for new combinations renders them invalid (Turland et al. 2018).

Abel Albert and Émile Jahandiez, in their Catalogue of the plants of Var, a département in Provence, south-east France, clearly stated that they had followed Rouy’s unusual concept of the term “forme” (Albert and Jahandiez 1908: xlii). Their combinations under that ranking are also therefore invalid (Albert and Jahandiez 1908: 4).

##### Note 3.

In a short note Giovanni Francesco Re (1773–1833) included the name of *Anemone latifolia* coined by Carlo Bellardi (1741–1826) as well as part of his unpublished description, stating that the species was from uncultivated fields around Nice. A search for specimens of Bellardi’s collection in his Herbarium in Turin (TO) did not locate any original material (Guglielmone pers. comm.). Many of his specimens have been either damaged or lost; however, there is a reference cited by Bellardi to the Swiss herbalist Dominique Chabrey (1610–1667): “Anemone latifolia ex coccineo phaenicei coloris, unguibus parvis subpallidus Chabr. sciagr. p. 462” [Anemone with broad leaves and Phoenician red-coloured flowers, with small paler claws]. Chabrey’s description includes an illustration of a simple flowered plant with eight tepals under that name, which can serve as the lectotype for the name *Anemone latifolia* Bellardi ex Re.

##### Note 4.

The Abbé Alexandre Pons included the statement “Selon nous, cette espèce comprend deux variétés ou races bien distinctes” (Pons 1883: lxxxii). Although his reference to “races” does not fit the use of the accepted terms in the terminology of botanical ranks according to Art. 4 of the ICN (Turland et al. 2018), the use of the term variété for variety is acceptable (Art. 4.1).

#### ﻿ Anemone hortensis var. pavonina

Taxon classificationPlantaeRanunculalesRanunculaceae

(Lam.) Gren. and Godr., Fl. France 1: 14 (1848)

BD56394C-5C89-5860-BAAC-F3A4D2F95A83

≡ Anemone pavonina Lam. Encycl. 1(1): 166 (1783) Lectotype designated here (Art. 9.3, 9.4): France? Without metadata “Herb. Lamarck” central multiple-tepalled flowering stem [P00282070] (Fig. 1D).
≡ Anemone coronaria var. pavonina (Lam.) Pers. Syn. pl. 2(1): 97 (1806).
≡ Anemone hortensis subsp. pavonina (Lam.) Arcang., Comp. Fl. Ital.: 5 (1882, see note below).
≡ Anemone hortensis [forme] pavonina (Lam.) Rouy and Foucaud, Fl. France 1: 48 (1893) (nom. inval. See note above under synonymy of A. hortensis).
= Anemone regina Risso var. duplex Risso, Flore de Nice: 6 (1844) Lectotype designated here (Art. 9.12): France, Alpes Maritimes, specimen labelled “Anemone regina N.” in Risso’s Herbarium, Flore du Département des Alpes-Maritimes, tome IV, folio 24 (lecto. P!); syntype: France, Alpes Maritimes, “Champs des collines”, Pointe Saint-Hospice, Cap Ferrat, *Risso s.n.* 1808 (syn. G-DC!) [G00144565].
= Anemone pavonina var. duplex Loret, Bull. Soc. bot. France 6: 33 (1859) Lectotype designated here (Art. 9.12): France, “Entre Cannes et Auribeau”, *H.Loret* s.d., s.n. Herb. Loret (lecto. P!) [P03181972]; syntype: France, “De Cannes à Auribeau” *H.Loret* 210 s.d. Herb. Loret. (syn. P!) [P03182118]; syntype: France “Cannes” *H.Loret* s.n. s.d. (syn. P!) [P00266525].
= Anemone hortensis var. multisepala Albert and Jahand., Cat. pl. vasc. Var: 4 (1908) Lectotype designated here (Art. 9.12): France, Var, “Bords des champs et des chemins, Montauroux. (Etiquette égarée = lost label)” *A.Albert* s.n., 15 March 1904 (P. lecto!) [P02695672]; (isolecto. LY x 2!) as “A. regina var. multisepala Herbier A. Albert” *A.Albert* s.n. [LY0030479; LY0030470]; (isolecto. MPU x 2!) [MPU522731; MPU522732]; syntypes: France, Var. Montauroux (as var. acutisepala) “sous les oliviers”, *A.Albert* s.n, 20 March 1904 (syn. P!) [P03181968]; Herbier A. Albert, Montauroux (Var), 20 March 1903, *A.Albert* s.n. (syn. LY x 2!) [LY0674586; LY0030469]; Montauroux 15 May 1906, leg. *A.Albert* s.n. (syn. LY!) [LY0030476]; Montauroux, 8 April 1903 *A.Albert* s.n. (syn. TLON!) [TLON07884]; syntype Callian (*Albert*) n.v.”.

##### Note.

In the Introduction to his “Compendio della flora Italiana”, Giovanni Arcangeli (1840–1921) stated, “Ebbi cura inoltre di enumerare e descrivere solo le forme specifiche principali e ben accertate, includendo nel numero delle sottospecie e varietà, e talora omettendo, quelle non poche di data recente ed ancora non sufficientemente studiate, delle quali varii dei moderni fitografi si sono compiaciuti arricchire la flora nostra. Le sottospecie o razze che si presentano in alcune specie sono state contrassegnate con lettere greche, mentre le varietà sono state distinte con lettere latine. [I have also taken care to enumerate and describe only the principal and well-established specific forms, including in the list some subspecies and varieties, and sometimes omitting, not a few of recent date and which are still not sufficiently studied, with which several modern phytographers have been pleased to enrich our flora. The subspecies or races which appear in some species have been marked with Greek letters, while the varieties have been distinguished with Latin letters.]” (Arcangeli 1882: vi). This statement whereby the Greek letter γ is placed before his entry for “22. *Anemone hortensis* γ *pavonina* validates it at the rank of subspecies even though he incorrectly attributes the basionym to Candolle but correctly states it to be a monstrous form (“forma mostruosa”) with “sepali molto numerosi, lanceolati o lanceolato-lineari, acutissimi” [multiple number of lanceolate or linear-lanceolate, most acute sepals] (Arcangeli 1882: 5). A year before his death he published a description of what he later termed Anemone hortensis var. pavonina, which clearly indicated that he was referring to multiple-tepalled plants that sometimes produced an outer ring of sepaloid photosynthetic bracts (Arcangeli 1920: 53).

##### Distribution and habitat.

Anemone hortensis var. pavonina is found across the range of the species and has become widely naturalised around Nice and along the Ligurian coastal region of Italy and also in the Landes region of Gascony (Fig. 1A) in southwestern France. It grows in open meadows, among shrubs, sometimes in woodland clearings from 100 to 1600 m. Flowering from March to May. Some double-flowered plants also occur occasionally among wild populations in Eastern Europe.

#### ﻿ Anemone hortensis var. stellata

Taxon classificationPlantaeRanunculalesRanunculaceae

Gren. and Godr., Fl. France 1(1): 14 (1847)

4AEF2A5C-14D2-56ED-AAD2-C2CBB654C8F3

Fig. 2A

Anemone hortensis var. stellata Gren. and Godr., Fl. France 1(1): 14 (1847) Lectotype designated here (Art. 9.3): France, Provence, Grasse, “Anemone stellata Lam., Grasse, *Mr de Baudot s.n.* 1837” with a label for “Herbier de la Flore de France de D. A. Godron” on the sheet (lecto. NCY!) [NCY0087945]; syntypes: France, Provence, “Anemone hortensis a stellata, Grenier 1846” with a label for Herbier de la Flore de France de D. A. Godron. *C.Grenier* s.n. (syn. NCY!) [NCY0087939]; France, Provence, Grasse, “Anemone stellata, Grasse, *Lenormand s.n.* 1839” with Herbier de la Flore de France de D. A. Godron label (syn. NCY!) [NCY0087940]

##### Distribution and habitat.

Anemone hortensis var. stellata (Fig. 2B) is found in Albania, Algeria, Bulgaria, Corsica, Croatia, Cyprus, France, Greece, Italy, Montenegro, North Macedonia, Sardinia, Sicily, Slovenia, Spain, and Turkey. It grows in open meadows, among low scrub, vineyards, sometimes in woodland clearings from 100 to 1200 m (c. 4000 ft.). Flowering from March to May. Teratological specimens also occur within this variety.

#### ﻿ Anemone hortensis var. heldreichii

Taxon classificationPlantaeRanunculalesRanunculaceae

(Boiss.) Halácsy, Consp. Fl. Gr.: 6 (1900)

382E2790-781C-59DE-97E1-30C9720F54CE

≡ Anemone stellata var. heldreichii Boiss. Diagn. pl. orient. ser. 1. vol. 2. fasc. 8: 1 (1849). “Hab. In dumosis montis Akrotiri et faucibus montis Malaxa prope Cydoniam, Cretae [growing in the thickets on the mountains of Akrotiri and the jaws of mount Malaxa near Kydon (Chania), Cretae (Kriti)]. *Heldreich* 1312, Marte 1846”. Lectotype designated here (Art. 9.12): Greece, Crete, (lecto. G!) [G00788416] Herb. G-BOIS. SIB no. 457133/1; isolecto: BM! [BM000613694]; isolecto. E! [E01097614]; isolecto. K x 3! [K000692042; K003266388; K003266412]; isolecto. LY [LY0796695]; isolecto. P x 4! [P00158570; P03182159; P03182319; P03182182]; isolecto. WAG! [WAG0043946]; isolecto. WU! [WU0074909].
≡ Anemone hortensis subvar. heldreichii (Boiss.) Hayek, Repert. Spec. Nov. Regni Veg. Beih. 30: 319 (1924).
≡ Anemone heldreichiana Gandoger, Fl. cret.: 6 (1916).
≡ Anemone hortensis subsp. heldreichii (Boiss.) Rech.f., Denkschr. Akad. Wiss. Wien, Math. -Naturwiss. Kl. 105(2,1): 743 (1943).

##### Distribution and habitat.

Anemone hortensis var. heldreichii is endemic to Crete, Karpathos and Kasos, occurring on mountain meadows and among rocks from 400 to 1200 m. Flowering February to April. Illustration Fig. 2D.

In addition to these varieties, it is worth noting a minor flower colour variant that occurs frequently in populations across the range of the species and which is recognised here as a new combination at the rank of forma. Plants with a pronounced zone of white, cream or pale yellow near the base of each tepal (Fig. 2C) can be assigned to Anemone hortensis f. regina (Risso) J.Compton. This is clearly evident in the three flowers on the specimen from Risso’s personal herbarium now conserved in Paris.

#### ﻿ Anemone hortensis f. regina

Taxon classificationPlantaeRanunculalesRanunculaceae

(Risso) J.Compton, comb. et stat. nov.

A52D0F59-7571-5CBC-9AC1-183E4CB09B28

≡ Anemone regina Risso, Flore de Nice: 6 (1844) Lectotype designated here (Art. 9.12): France, specimen annotated “Anemone pavonina? Risso cat. pl. ind. Alp, mar. p. 403 (1826), donnée par Mr. Geny en 1860” and with a label ‘Herbier RISSO, constitué dans les Alpes-Maritimes vers 1820–1860 reçu au Muséum en 1955. HERB. MUS. PARIS’ (lecto. P!) [P02467501] (see note below and Fig. 2C).
≡ Anemone pavonina var. regina (Risso) Gürke, Pl. Europeae 2 (3): 468 (1903).
≡ Anemone hortensis [forme] regina (Risso) Rouy and Foucaud, Fl. France 1: 49 (1893) nom. inval.
(≡ Anemone hortensis [forme] regina (Risso) Albert, Cat. pl. vasc. Var: 4 (1908) nom. inval.).
≡ Anemone hortensis var. regina (Risso) P.Fourn., Quatre Fl. France: 348 (1936).
= Anemone hortensis var. ocellata Moggr., Contr. Fl. Mentone ed. 3. t. 1 (1874). Lectotype designated here (Art. 9.12) [icon] Contr. Fl. Mentone ed. 3. t. 1 (1874). Right-hand illustration.
≡ Anemone pavonina var. ocellata (Moggr.) Bowles and Stearn, J. Roy. Hort. Soc. 73(3): 69 (1948).

##### Note.

Phillipe Geny (1809–1875) settled in Nice in 1833 as inspector of plantations in that city. He was a close correspondent of Antoine Risso (1777–1845), providing all the rather inadequate illustrations for Risso’s “Flore de Nice” (Risso 1844), including a barely recognisable one of *Anemone hortensis* (as *A. stellata*), but curiously did not provide any illustration of Risso’s new species, *A. regina* Risso.

## ﻿Epilogue

This paper exemplifies problems that can occur when the protologue and its associated original material have not been correctly identified. As already explained in the case of Lamarck’s material of *Anemone pavonina* Lam., it has only relatively recently been possible to examine his herbarium in Paris and therefore verify what his type material is. A thorough examination of the original material of *Anemone hortensis* in BM, from two cultivated red-flowered specimens now known to occur in the wild in southeast Europe and southwest Asia, has likewise confirmed the identity of that species. These original specimens, recognised as A. hortensis var. hortensis, have ten, eleven and twelve broadly obovate tepals, each tepal with five main veins. In contrast, three further specimens of Linnaeus’s original material, two in LINN and one in S, can now be referred to as Anemone hortensis var. stellata Gren. and Godr. The latter specimens have flowers with thirteen, fifteen and sixteen narrowly lanceolate tepals, each with three veins. In the wild these consistently have lilac or purple flowers. Plants endemic to Crete and nearby islands, with white or rarely pinkish flowers and twelve to twenty elliptic three-veined tepals, are recognised as A. hortensis var. heldreichii (Boiss.) Halácsy.

The results shown here, accumulated from a number of different sources, all conclude that samples of *Anemone hortensis* and *A. pavonina* belong within a single species which exhibits only minor levels of genetic and morphological variation. Three persistent morphological variations at the rank of variety and an additional minor flower variant at the rank of forma are proposed here for long-term nomenclatural use and consistency.

## Supplementary Material

XML Treatment for Anemone hortensis

XML Treatment for Anemone hortensis var. pavonina

XML Treatment for Anemone hortensis var. stellata

XML Treatment for Anemone hortensis var. heldreichii

XML Treatment for Anemone hortensis f. regina

## ﻿Additional information

### Use of AI

No use of AI was reported.

### Data availability

All of the data that support the findings of this study are available in the main text.
